# The network limits of infectious disease control via occupation-based targeting

**DOI:** 10.1038/s41598-021-02226-x

**Published:** 2021-11-24

**Authors:** Demetris Avraam, Nick Obradovich, Niccolò Pescetelli, Manuel Cebrian, Alex Rutherford

**Affiliations:** 1 Centre for Humans and Machines, Max Planck Institute for Human Development, Berlin, Germany; 2 Population Health Sciences Institute, Newcastle University, Newcastle, UK

**Keywords:** Infectious diseases, Computational science

## Abstract

Policymakers commonly employ non-pharmaceutical interventions to reduce the scale and severity of pandemics. Of non-pharmaceutical interventions, physical distancing policies—designed to reduce person-to-person pathogenic spread – have risen to recent prominence. In particular, stay-at-home policies of the sort widely implemented around the globe in response to the COVID-19 pandemic have proven to be markedly effective at slowing pandemic growth. However, such blunt policy instruments, while effective, produce numerous unintended consequences, including potentially dramatic reductions in economic productivity. In this study, we develop methods to investigate the potential to simultaneously contain pandemic spread while also minimizing economic disruptions. We do so by incorporating both occupational and contact network information contained within an urban environment, information that is commonly excluded from typical pandemic control policy design. The results of our methods suggest that large gains in both economic productivity and pandemic control might be had by the incorporation and consideration of simple-to-measure characteristics of the occupational contact network. We find evidence that more sophisticated, and more privacy invasive, measures of this network do not drastically increase performance.

## Introduction

Containment of infectious disease requires implementation of strategies to reduce the spread of the disease^1^. Until a robust vaccine is widely available, as was the case for many months of the COVID-19 epidemic, the leading Non-Pharmaceutical Intervention (NPI) is physical distancing including closure of businesses^2^: a reduction of the number of people in physical proximity.

Since the European Industrial Revolution workers have increasingly migrated to towns and cities to convene in centralised workplaces^3^. A large literature of work relates growth, creativity and other metrics of economic performance directly to the increasing returns and economies of scale made possible by the density and proximity of workers in regions, cities and workplaces^4–6^. Therefore, physical distancing is unavoidably disruptive to these workers, workplaces and economies and is likely to cause long term shifts in working patterns. The highly infectious nature of COVID-19 along with the disease’s long incubation period^7,8^ have shown the cost of such disruption even more starkly than previous epidemics. As a result, working hour losses globally could be as high as 12% in the third quarter of 2020^9^ and a 6.6% drop in global GDP in 2020^10^. For this among other reasons, scholars have noted that the COVID-19 epidemic has the potential to affect the future of work substantially over the long term^11,12^.

More precisely, physical distancing policies affect *workplaces* directly and workers indirectly. While some work environments are conducive to physical distancing (e.g. construction sites), others are inherently not (e.g. gyms & nightclubs)^13^. As a result, physical distancing requirements have produced heterogeneous effects on workers that are primarily determined by workers’ ability to physically distance in their workplace and/or to work from home (WFH)^14^.

Thus policymakers have the difficult task of moving beyond coarse economic interventions e.g. furloughing all workers, to more targeted interventions that balance the human toll of infection with the economic cost of interventions. Stated precisely, workers should be partitioned into groups who may respectively (i) remain working in their workplaces (ii) work effectively from home or (iii) be furloughed, such that the spread of the epidemic is minimised while also minimising economic disruption associated with the intervention.

This optimisation task is currently impossible on a practical level for many reasons. These include the lack of sufficiently detailed information on human mobility and social interactions, the complex network dynamics of disease spread^15^, the interconnected nature of modern economies^16–18^, and divergent opinions on the proper balance between human and economic loss^19^.

Epidemics typically pose differing levels of risk to individuals based on demographic categories, such as age groups. This may be due to characteristics of the specific disease or due to differing physical contact patterns encoded in contact matrices^20^. This heterogeneity in the spread between members of a population also contributes to the overall complexity of accurately modeling the disease spread.

It is notoriously difficult to measure, at scale, physical proximity as it relates to airborne or droplet mediated transmission of infectious disease^21,22^. Deriving a *network* of contacts is even more challenging compared to a simple count of the number of contacts. This is due to privacy concerns and limitations of measurement accuracy as individuals need to be uniquely identified throughout the measurement^23^. Typically contact interactions are measured through self-reported surveys and networks measured using sensors^24–28^. Previous work has constructed contact matrices between subpopulations stratified by age and other demographics^29,30^. Full contact networks have been measured in large scale field studies in schools^31,32^, dormitories^33^, hospitals^34^, and conferences^35^ as well as being approximated from the use of location-based services^36^.

Yet, demographics represent only one set of individual characteristics that likely play a role in modulating differential epidemic spread. An individual’s work – their occupation – encapsulates many determinants of their epidemic risk. Commuting patterns, the extent of contact with others at their work, and the ease with which non-pharmaceutical interventions such as wearing of personal protective equipment and remote working can be adopted are all occupation-specific factors that shape both individual risk and the propensity for individuals in any given occupation to shape epidemic spread on human networks more generally.

While studies have measured contact networks in workplaces^37^, to date we are not aware of any in-depth study on contact networks stratified by occupation, and as such the true structure of such a network is unknown.

In this work, we examine the role of occupation in an epidemic spread in an urban environment. We also evaluate the efficacy of occupation-based disease control measures within a simulation of epidemic dynamics using the Susceptible-Exposed-Infected-Recovered (SEIR) framework. More specifically, we investigate the effects of several network-based interventions and compare them to the outcomes produced by more coarse heuristics. A focus on occupational interventions, coupled with detailed data on the distribution of the workforce across occupations, wage and workplace proximity allows us to simulate the economic impact of particular containment strategies alongside each intervention’s epidemiological impact.

Our methods enable us to approximate the degree of physical contact between individuals within and across occupations without explicit contact matrices (such matrices to our knowledge do not exist across the full empirical network due to the cost and methodological complexity of measuring such contacts). Our approach is general and applicable to any infectious disease, however, we parameterise our simulations with estimations of the epidemiological characteristics of COVID-19 for illustrative purposes.

Our work complements a rapidly growing body of work analysing the effects of physical distancing and furloughing policies on human mobility and behaviour in the COVID-19 epidemic^13,38–46^ with several focusing on the economic aspects in particular^47,48^.

Using these methods, we make three marked contributions to the science of epidemic control. First, we contribute a technique for constructing occupation-based epidemic simulations using publicly available data. Second, we compare several occupation-based containment policies based on their epidemiological and economic costs. Third, having identified contact degree as an effective heuristic for containment, we extend to investigate the fundamental limits of the controllability of epidemics in networks. Our findings have implications, not only for the containment of future epidemics, but also for the future of work and the interaction between disease risk at work, automation risk and the economics of labour.

## Data and methods

We present a general method for simulating occupation-based epidemic control policy interventions. However, we consider data from New York City as a paradigmatic example^49^. We make use of the Occupational Information Network (O*NET) which is a public repository of occupational data in the US and statistical economic information collected by the Bureau of Labour Statistics (BLS).

### O*NET: occupations and work characteristics

**Figure 1:**
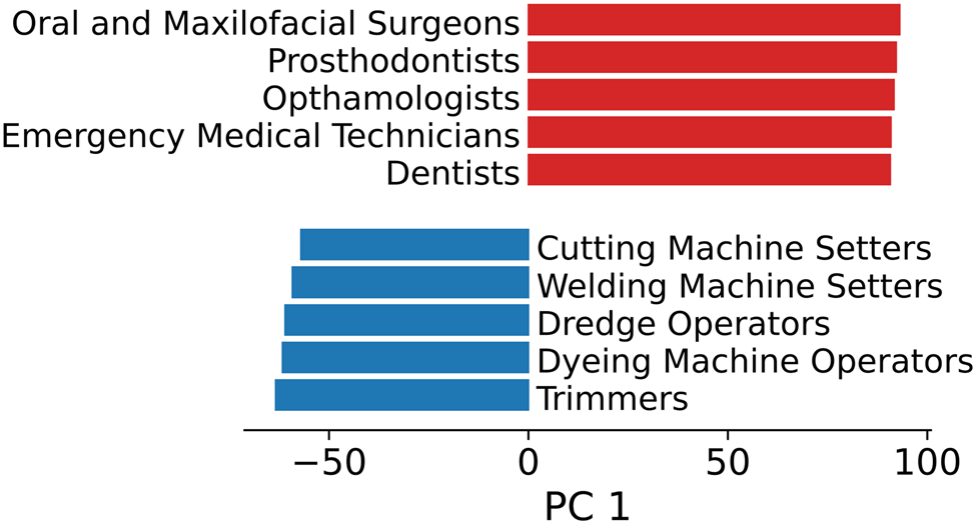
The five occupations with largest and smallest projection onto the first principal component of proximity measures. The PCA operates on five independent measures of proximity required by each job taken from the O*NET database: *Exposed to disease or infections, Performing for or working directly with the public, Communicating with persons outside of the organisation, Deal with external customers, Physical proximity*. The first Principal Component can explain 53% of the total variance. See  SI for more details.

We make use of the O*NET data on “work characteristics” to derive a composite measure of proximity from five distinct work characteristics that are likely to be correlated with the degree of in-person contact a worker is required to have. These are Exposed to disease or infections, Performing for or working directly with the public, Communicating with persons outside of the organisation, Deal with external customers, Physical proximity. We construct a composite measure of proximity from these five dimensions using a Principal Component Analysis (PCA). We inspect the occupations that are assigned high and low degree to manually validate this method (see SI). The projection on the first principal component (PC) is listed in Fig. 1 for the top/bottom five jobs. We note that this first order approximate measure excludes certain dimensions that are likely to be important in preventing disease transmission in work environments such as the ability to incorporate protective measures into the workplace.

### Contact data: proportions of home/work/transit

In agreement with previous empirical studies of contact networks^50^, we define three categories of contacts among the adult working population: home, work and transport. The mean total contact degree has been found to be 75 in a recent study of proximity via Bluetooth in New York City^51^. Fixing the mean home degree at two based on the mean household size^52^, and considering an equal work and transportation mean degree, we set the ratio of the mean contact degree to be 4:73:73 for home:work:transport, respectively (see SI). We fit the empirical data found in^30^ to derive a log-normal distribution for each category rescaling the mean to fit the above ratios. For each worker node, we sample each of the three distributions to determine total degree. The number of work contacts for each worker depends on her occupation, while the number of home and transportation links are independent of occupation. Consider a worker in a high proximity occupation such as a Retail Salesperson, links are assigned as follows:

- The proximity score of the occupation is taken as the projection of the job on the first principal component derived from the PCA of the proximity variables. For the case of a retail Salesperson this is 0.55 in the range [0-1].
- This specific PCA value is mapped to a log-normal distribution (mean of 36.5) of work degree by mapping the percentiles of the distributions yielding a work degree of 49.
- A transport degree is assigned to the worker node by independently sampling a log-normal distribution with a mean of 36.5 and a standard deviation of 25.55 (see SI).
- A home degree is assigned to the worker by independently sampling a log-normal distribution with a mean of 2 and a standard deviation of 1.4.

Links from each category are functionally equivalent in terms of disease transmission in our simulations. However each layer is independent, and different policy interventions retain or remove links from each layer as described later.

### Work from home data

Some workers can work from home without disruption to productivity and thus may be effectively removed from the occupational contact network without loss of economic contribution. We consider a binary index for O*NET jobs derived by Dingel and Neiman^14^.

### Essentialness data

Since one motivation for NPI selectively furloughs workers according to perceptions of the essentialness of their contributions, we assign a measure of ‘essentialness’ using the data of del Rio-Chanona et al (2020)^47,53^.

### Wages data

Another NPI we consider here to balance between the loss of economic productivity and the epidemic expansion is the furlough of workers prioritising those on a low income. We use wage data by occupation for New York, based on the Occupational Employment Statistics survey by US Bureau Labor Statistics^54^. For most of the occupations, the dataset includes the average annual wages while for hourly-paid occupations like actors the dataset includes the mean hourly wages. For both cases, we estimated the average daily wage per occupation.

### Construction of contact network

We construct the contact network as follows.

**Figure 2:**
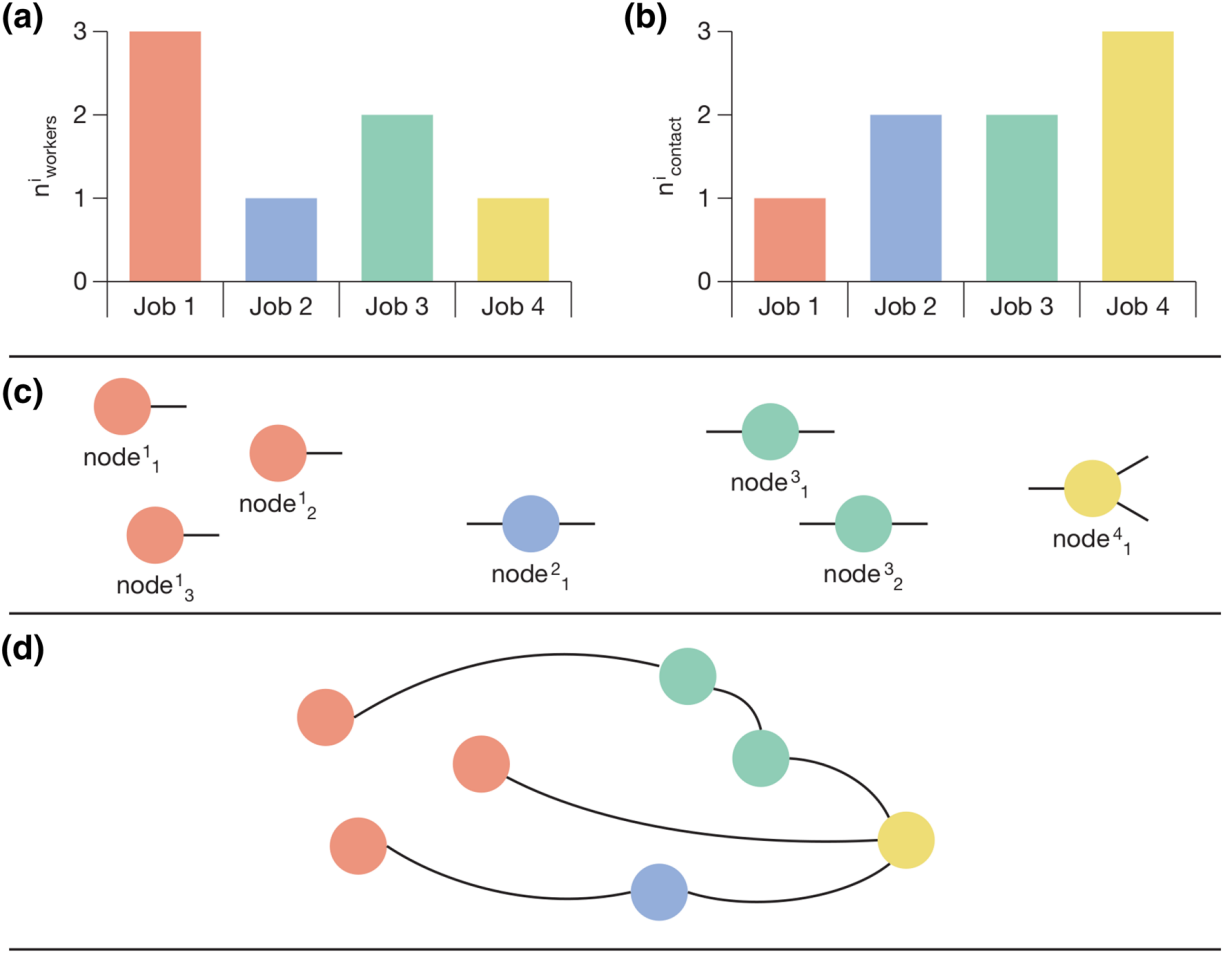
Assignment of work contact links to nodes representing workers and construction of contact network using configuration model procedure. Clockwise from top left (**a**) worker nodes are created according to the empirical distribution in the workforce (**b**) work contacts are assigned according to the occupation wise proximity index (**c**) half-links are attached to nodes according to work contact degree (**d**) pairs of half-links are joined at random to form a full network. Home and transportation degrees are sampled from a distribution on a worker basis and, unlike work degree, are not determined by occupation.

1. We define N=200,000 representative workers each with a specified O*NET occupation as nodes in our contact network. The proportion of workers in each occupation matches workforce data for New York City. The total number of workers is the minimum number that allows for at least one worker of each occupation.
2. Each worker is assigned a contact degree for home, work and transport. The work contact degree is determined by the proximity score for the worker occupation. Home and transport contact degrees are drawn from fixed distributions independent of occupation (see SI).
3. The nodes are connected to form the contact network using the configuration model^55^ (see Fig. 2).

The synthetic networks produced by this approach have some resemblance to a configuration model. However, the configuration model typically samples from a degree distribution to assign stubs to nodes, whereas in our case the node degree sequence is known and determined by the distribution of occupations in the workforce.

Each node has as many half links as the number of home, work and transportation contacts of the worker represented by the node. Pairs of half links for each category of contacts are then connected randomly until all half-links are paired up generating the final contact network. We conduct epidemic simulations in one realisation of the contact network constructed by the configuration model. In SI, we repeat the analysis in a second realisation of the contact network drawn from the same configuration model. We make the assumption that the contact network is fixed throughout the epidemic period. While this is unrealistic, the changing patterns of physical colocation are prohibitively complex to model as to be out of scope of this paper.

### Epidemic simulation

Our simulation of worker based epidemic spread and containment proceeds as follows.

1. We construct an SEIR model^56^ in which each worker may be in one of the four epidemic compartments. At the initial time step all nodes are considered as susceptible. An infection is seeded with one randomly chosen infected node.
2. Once the epidemic has reached 200 nodes (i.e. 0.1% of the synthetic population), we implement one of the intervention strategies described below. Epidemics that terminate before reaching 200 nodes are discarded.
3. The simulation continues in daily time steps until the epidemic terminates.
4. For each epidemic simulation, we calculate the severity of the epidemic and the economic cost of furloughing workers and workers who are unproductive due to illness.

### Epidemic parameters

We emphasise that our methodology is designed for infectious disease epidemics in general. In this case we parameterise our SEIR simulations using the epidemic parameters within the reported ranges for the COVID-19 virus (see Table 1).

We make use of a standard SEIR model in which each worker is assigned to an epidemic state. Specifically, during the epidemic process, each node is in one of four states: susceptible (S), exposed (E), infected (I), and recovered (R). A susceptible node will become exposed for a certain period after being infected, and will transit into the infected state with a certain probability. At each time step, an infected node can recover with a fixed recovery rate. Both exposed and infected nodes can transmit the disease to a susceptible neighbor in the network with the same infection rate.

With a fixed basic reproduction number, $R_0$ (that is, the average number of secondary infections an infected individual causes before the individual recovers) and given the average degree, $\langle d \rangle$, of our synthetic contact network, we derive an estimated transmission rate per contact (see SI)

$$\begin{aligned} p_{inf} = 1 - (1 -( R_0 \langle d \rangle )^{(1/(\langle t_{recovery} \rangle + \langle t_{incubation} \rangle ))}) \end{aligned}$$

with the mean-field approximation assuming that each node has the same connectedness equal to the mean degree and recovers in $\langle t_{recovery} \rangle + \langle t_{incubation} \rangle$ days if the individual is infected.

**Table 1:** COVID-19 epidemic parameters.

$\langle R_0 \rangle$	2.5^57–59^
$\langle t_{recovery} \rangle$	14 days^60^
$\langle t_{incubation} \rangle$	5.1 days^61–63^

Each epidemic simulation is seeded with one randomly infected node. Once the epidemic grows to more than 200 infected nodes, we implement a policy intervention into the network. If the epidemic dies out before reaching 200 nodes then the simulation is discarded, as we are here interested in modelling only those critical scenarios where human or policy interventions may be important factors in affecting epidemic outcomes.

### Intervention strategies

We seek to find the dually optimal strategy – both in infection control and economic terms – that a policy maker might use to contain the epidemic. This requires leveraging occupation-specific information to selectively remove worker nodes from the contact network, i.e. furloughing workers. This must be done in such a way that both the severity of the epidemic, as measured by the peak of the number of infected workers, and the economic cost of furloughing workers is jointly minimised. In our simulation study, we furlough workers based on the intervention strategies shown in Table 2.

**Table 2:** Occupation-based intervention strategies.

Strategy number	Name	Description	Note
$-$1	Baseline	No workers are furloughed. Epidemic proceeds uninhibited.	
0	Work From Home	All workers who can work from home have work and transport links cut.	40% of workers are able to WFH^14^
1	Random	In addition to those who can work from home (strategy 0), send home n% of remaining workers at random.	
2	Most connected	Additionally to strategy 0, send home n% of remaining workers ordered by contact degree (from highest to lowest).	
3	Least Essential	Additionally to strategy 0, send home n% of remaining workers ordered by ‘essentialness’ (from lowest essential score to highest).	
4	Cheapest	Additionally to strategy 0, send home n% of remaining workers ordered by wage (from lowest to highest).	
5	Centrality	Additionally to strategy 0, send home n% of remaining workers ordered by network centrality (from highest to lowest).	We consider various centrality metrics (see SI) and report the best performing HDA
6	Control	Additionally to strategy 0, send home n% of nodes ordered by degree, prioritising control nodes.	We consider the Switchboard model^64,65^. We find 47.9% of nodes are identified as control nodes

### Economic productivity

Approximation of the economic impact of a worker unable to work either due to infection or furlough is a challenging measurement task^66^. We investigated two metrics: (i) the average wage of the furloughed/infected worker and (ii) the contribution to macro-economic productivity derived from the occupational share of the larger industry-level productivity. Due to the coarseness of the best available data (and the subsequent risk of substantial measurement error) for (ii) (see SI) we proceed with measure (i).

The total cost of lost productivity of infected workers is calculated as the sum of the daily wages over the period that each worker is infected (the daily wage of each worker is based on his/her occupation). The total cost of workers who are furloughed is calculated as the sum of their daily wages over the furloughed period (the furloughed period is the difference between the length of the epidemic period and the length of the intervention period).

In the former case (wage-based calculation), the wage of a worker is considered proportional to her economic contribution and her loss of income as a loss in disposable income that can be spent to stimulate supply. We acknowledge that wage is an imperfect measure of the economic contribution of a job. However we conclude from the best quality data on industry level economic contributions that estimation is not possible (see SI). Results in the main paper correspond to (i).

### Ethics declaration

All analyses including human data followed relevant ethical guidelines.

## Results

**Figure 3:**
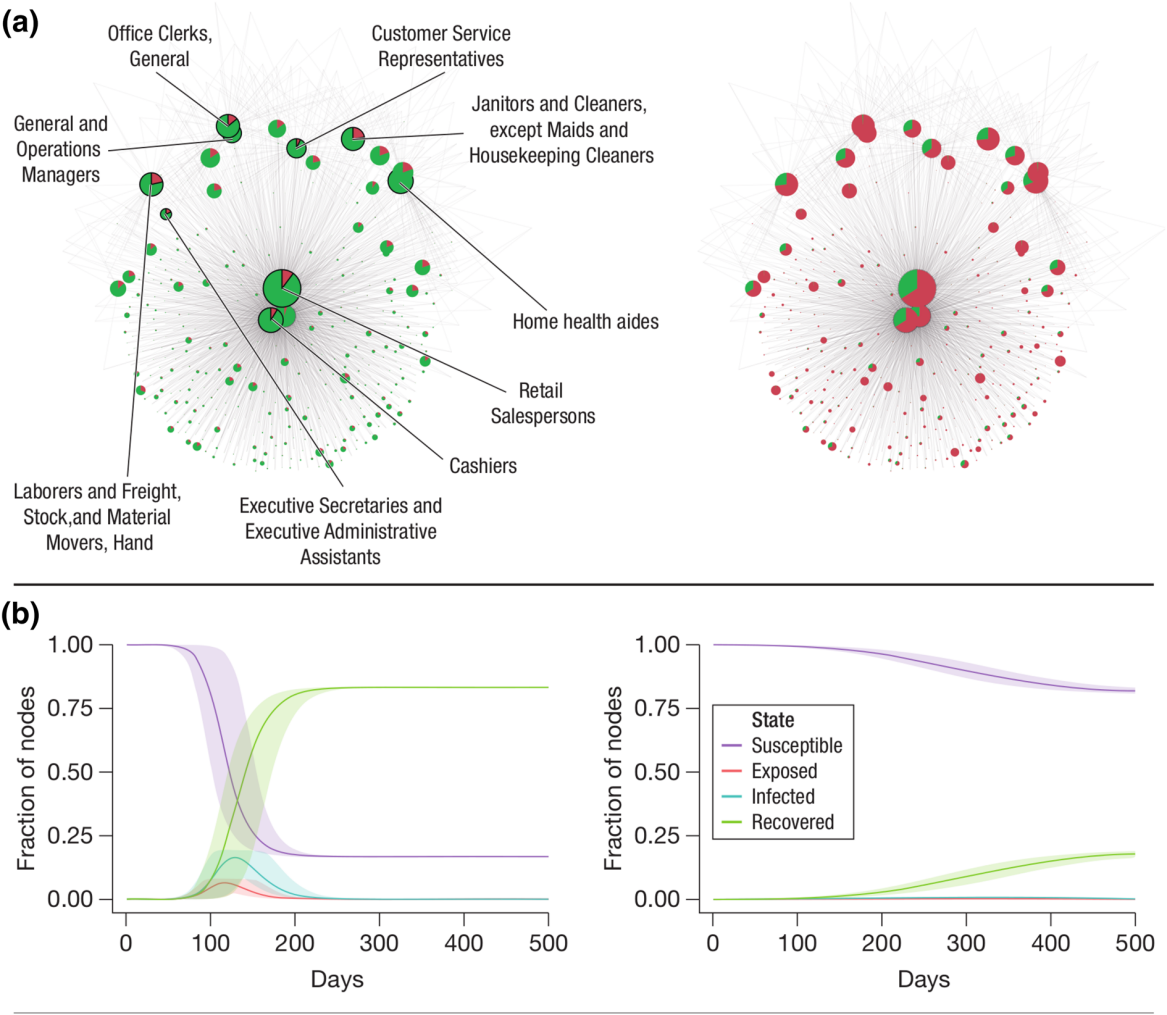
Epidemic dynamics under ‘strategy 0: no intervention’ (left plots) and ‘strategy 2: in addition to those who can work from home, send home n% = 10% workers based on highest degree’ (right plots). Top row (**a**) shows workers aggregated into occupations with the node size proportional to the number of workers and coloured by proportion of susceptible (brown) and recovered (green). Edge between two nodes indicates contacts between workers from the two occupations weighted by the number of such contacts (for visualisation purposes we only display the top three by weight edges for each node). Bottom row (**b**) shows the mean of 1000 epidemic curves and 95% confidence intervals.

Figure 3 presents a comparison between the ‘no intervention’ and ‘furloughing 10% based on degree’ strategies, respectively. Under an uninhibited outbreak (strategy -1) the peak of infected nodes occurs at a mean of 129 days and with a mean 19% of nodes infected which provides reasonable correspondence to observed outbreak dynamics. This changes to a mean of 164 days and 6% infected respectively under the strategy in which workers in occupations that can work from home have transport and work ties cut. Due to the difficulty in meaningfully representing the full contact network, we have instead represented the full network aggregated to  ~ 700 nodes representing occupations while still capturing both (i) the number of workers in each occupation (the node size) and (ii) the proportion that are infected (the proportion of the node colour).

**Figure 4:**
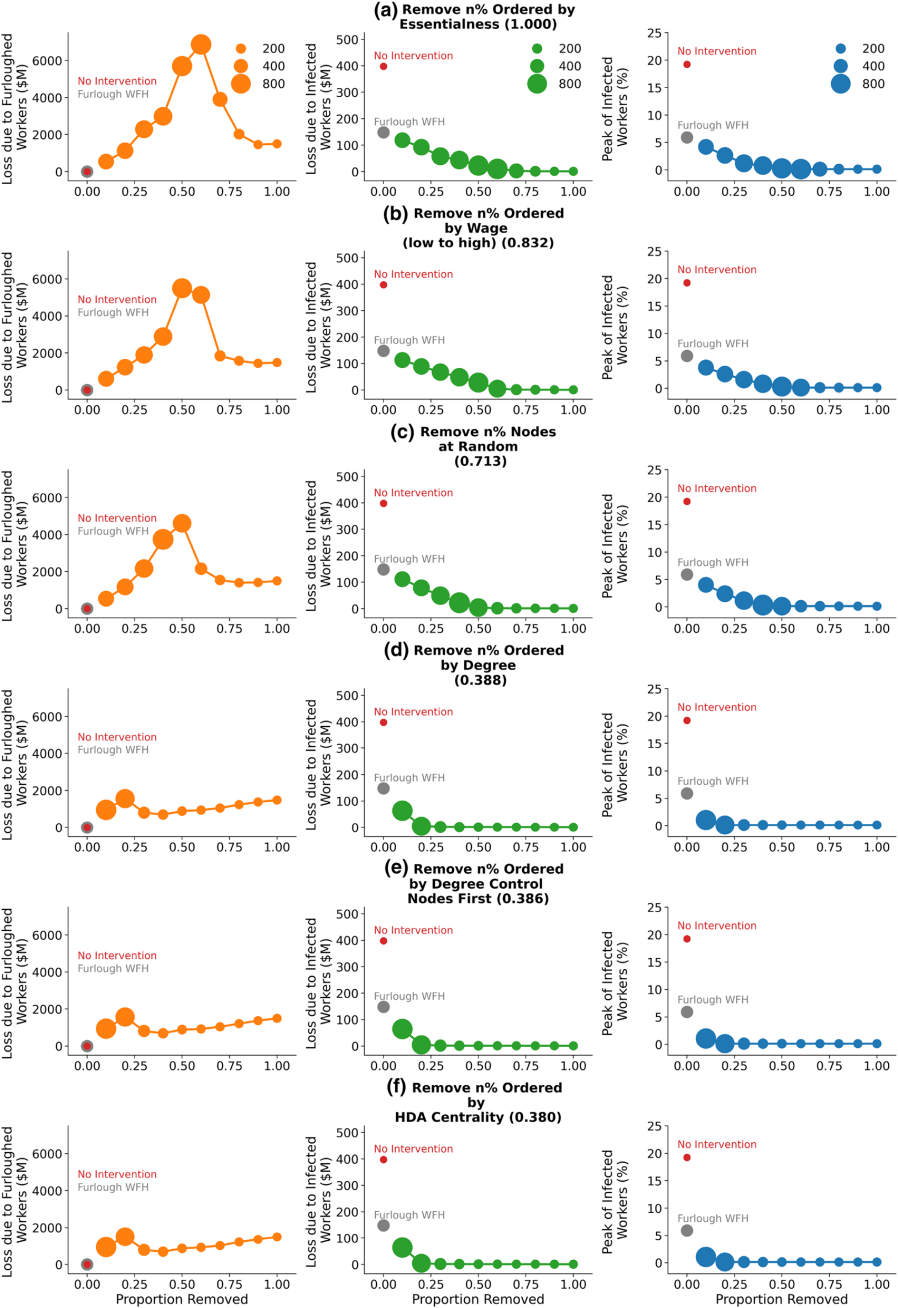
Comparison of occupation furloughing strategies ordered by economic cost of furloughing (the cost is indicated in brackets next to the strategy description relative to the worst performing strategy). The total wage loss due to furloughing (left column), wage lost due to infection (middle column) and proportion of workers infected at peak of epidemic curve (right column) as a function of the proportion of workers removed. The fixed points for null strategies of no intervention (red point) and furloughing only workers who can work from home (grey point) are marked for comparison. Markers are sized by the duration of the epidemic period in days, defined as the time for the number of infected and exposed workers to drop to zero. Each point is an average over 1000 epidemic simulations.

Figure 4 compares each strategy as a function of the stringency of the strategy (percentage of workers furloughed). In particular, the economic cost of furloughing workers calculated from wage (left) the economic cost of infected workers (middle) and the size of the peak of infected workers (right). We consider these distinct measures of epidemic severity as independent measures that policy makers would like to jointly minimise. The strategies are ordered in terms of total cost of furloughing across the range of stringency; most to least expensive, top to bottom. Generally, the measured economic loss due to infection is an order of magnitude less than the loss due to furloughing, since the infection period is ~ 14 days while the furlough period is the duration of the entire epidemic process (from the day that the intervention is triggered until there are no infected or exposed individuals); it is cheaper to allow a worker to be infected than to pay her wages for the duration of the epidemic.

We acknowledge that epidemic control is a careful balance of economic and human losses that is determined by some unknown and subjective multiplier of the pure cost of wages of infected workers due to secondary effects of infection e.g. lasting after effects of the disease, deaths caused by the disease, loss of quality years of life, psychological effects and so on. Likewise, furlough has negative secondary effects on individual workers^66,67^. Further, we recognise that the implementation of these strategies would be a logistic challenge that might favour a lower level of stringency. The range of severity of mobility restrictions has varied vastly between countries, from a laissez-faire approach (Sweden) to a ‘COVID-zero’ strategy warranting total lockdown (New Zealand). Our rationale is to thoroughly explore the behaviour across the phase space. For this same reason we do not attempt to directly compare losses on these three distinct dimensions or to prescribe the extent to which workers should be furloughed. Instead we seek to compare the general behaviour of various interventions in terms of the complex dynamics of the contact network, labour market and epidemic dynamics. We also note that the economic cost includes both the case of the state bearing the cost of covering the salaries of furloughed workers i.e. a strong social safety net and also the case that the worker herself must use savings, family support etc to replace lost wages. We consider the performance of each strategy across the entire range of severity of the strategy implementation i.e. the percentage of workers who are furloughed.

The loss due to furloughing shows a peak, at around 50% of removed nodes, for the worst performing strategies (left column rows a-c). This is related to the duration of the epidemic process (the size of the plot markers) as workers are furloughed until the epidemic process terminates. When an intermediate number of workers are removed, the epidemic is still able to spread but takes longer to fully terminate. Note that this is not attributable to herd immunity as the proportion of recovered nodes remains well below the 70-85% range. The better performing strategies (rows d-f) remove nodes more effectively, causing the epidemic to terminate sooner. In contrast to the other strategies, removing more than ~ 30% of nodes based on controllability, centrality and degree strategies (d-f) does not cause the epidemic period to increase significantly. This leads to a roughly linear increase in furlough costs as more workers are furloughed for the comparable duration.

In contrast, the loss due to infected workers (middle column) decreases monotonically; the total number of infected workers decreases as network edges are cut. Likewise the size of the peak of the infection (right column), representing the strain on health facilities, decreases monotonically as the ‘curve is flattened’.

It should be noted that the varying behaviour of the strategies is generally not trivially attributable to the breaking apart of the underlying worker network, particularly as furloughing cuts only work and transport links preserving home contacts analogously to a home quarantine (see SI). For each strategy, the size of the Largest Connected Component decreases at roughly the same rate and contains no less than 80% of nodes across all strategies and the range of severity. Our results are broadly unchanged when home links are also severed i.e. a centralised quarantine (see SI).

We find that removing workers according to essentialness underperforms even random node removal. This is consistent with only a moderate correlation ($\rho = 0.19$, see SI) between our proximity score and essentialness score; non-essential workers do not necessarily have a high contact degree and so their removal still allows efficient epidemic spread. Likewise removing based on wage alone performs poorly, consistent with a weak correlation between wage and proximity ($\rho = 0.05$, see SI). For example, construction or mechanical occupations have low proximity to others. As a result, the epidemic can spread quite effectively until a large proportion of workers are furloughed (n% $\sim$ 100%).

**Figure 5:**
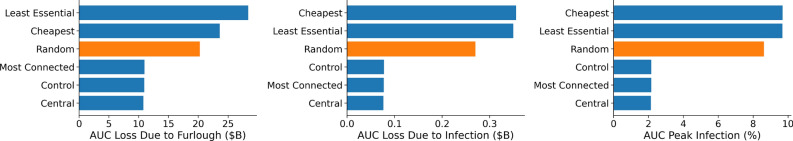
The aggregated performance of each strategy across the full range of severity of implementation for each measure; cost of furloughing workers (left), cost of infected workers (middle) and peak of infection (right). We recall that the lower the curves in Fig. 4, the more optimal is the strategy for reducing cost on all dimensions. Therefore we use the Area Under the Curve (AUC) as an aggregated measure of how each strategy performs. Error bars representing 95% confidence intervals are no greater than 0.7% of the mean value and so are omitted here for clarity.

Removing workers based on contact degree shows a drastic increase in performance (38.6% of economic cost of the worst performing strategy; removing nodes according to essentialness) when considering the cost of infected workers. Notably, the best performing centrality metric is almost indistinguishable from node degree (38.6% vs 38.6%). Despite centrality metrics incorporating full knowledge of the network structure, they were unable to halt the spread of the epidemic markedly more effectively than simple knowledge of a workers local environment. Likewise for the controllability based strategy (38.6% vs. 38.0%). Figure 5 evaluates the overall performance of each strategy across the range of stringency (n%). We repeat our analysis using an alternate random network instantiation created using the same methodology as described above. We find our results are robust to these alternative specifications (see SI).

Control nodes of a network are defined as the nodes which, if their state can be manipulated, allows for the state of the entire network to be driven to a desired configuration. Under the model of switchboard controllability^68^ (considered the most appropriate model for epidemics), we find that 47.9% of nodes in our network are identified as control nodes. Such a large proportion implies that the contact degree has a low capacity to drive controllability, which is consistent with previous studies of social networks^68^ in comparison to more explicitly hierarchical networks such as those belonging to organisations or neuronal structures. Consequently, we find that a controllability based strategy offers no significant improvements in performance over a degree based strategy. This is despite incorporating full knowledge of the network structure.

These empirical results illustrate the difficulty of fully controlling an epidemic ex-ante, even with the advantage of full information of the contact network and justify the use of manual^38,59,69^ and digital contact tracing^70,71^ for containment as a local and dynamic strategy (as opposed to static and global one). Correspondingly, we find that removing control nodes preferentially is not a strategy that performs well, which suggests that the dynamics of epidemics might not amenable to network controllability^68^.

Given that our contact network is not empirical, it is possible that our results arise as an artifact of how the network was constructed from occupational characteristics. We emphasise that to our knowledge there does not exist a publicly available contact network larger than $\sim 10^2$ nodes, and none of any size that considers occupational subpopulations, that could be employed directly or bootstrapped against. Nevertheless we compare our generated network to a number of smaller empirical networks from different contexts including workplaces, schools and conferences (see SI). We find congruence between most metrics excluding those that were not explicitly coded in our configuration model such as transitivity and assortativity. This includes the high density of control nodes, in the range 40-50%.

## Discussion

In this paper we have described a method to construct epidemic contact matrices between detailed occupational sub-populations using publicly available data. We use these contact matrices as input to SEIR simulations in an urban environment and compare various epidemic containment strategies in terms of their economic and human cost. We emphasise that our goal in this paper is not to predict the spread of COVID-19, but rather to compare various occupation-based containment strategies more generally using the COVID-19 epidemic as a motivating case. We find that the heuristic of worker node removal according to network degree (the number of physical contacts that a worker has) performs approximately the same as more complex metrics based on complete network structure or other occupational characteristics. More broadly we note that epidemic contact networks exhibit low levels of controllability.

Our findings demonstrate that the structure of the contact network heavily influences disease dynamics in non-trivial ways. For example, furloughing a small proportion of workers can lead to pruning of the network in such a way that the epidemic persists for a long time, albeit at low levels, leading to a long and costly furlough. Intuitive strategies such as furloughing workers based on the essentialness of their job, by wage or at random all perform poorly on this basis. In contrast, network-based metrics such as degree and centrality are able to reduce the peak of the infection (flattening the curve) and also reduce the epidemic period.

Our findings have been shown to apply in New York City; a large, dense and wealthy city with a diverse workforce. We would expect that other dense megapolises with widespread use of public transport to share similar mixing patterns. However the distribution of occupations are likely to be very different depending on urban economic specialisations. In general, larger cities support more diverse job markets, and vica versa for smaller cities. Therefore we might expect more homogeneity in the contact degree across workers in smaller urban areas and rural areas, this would lead to lower efficacy in degree based strategies. Likewise intra-household mixing is likely to play a larger role when cultural and economic constraints lead to larger family units.

Our methods exist in a relative paucity of sufficiently rich data to simulate real-world dynamics with high fidelity. Empirical contact networks, whether self-reported or passively sensed, are only freely available on a small scale. Networks with any demographic information are rarer still, while contact networks with occupational information are not to our knowledge available at all. In a workplace the correspondence between physical contact and social ties is likely to be weaker as some jobs, particularly service jobs, require a great deal of face to face contact. Consequently social ties are likely not to be a trivial proxy for physical contact ties, as observed previously^36^. Thus social networks are not a viable proxy for worker contact networks. In general we find that occupational attributes relevant to epidemic containment (proximity, essentialness) are only weakly related to wage. As a result there is a non-trivial relationship between occupational characteristics and epidemic dynamics that make the dual optimisation of economic and human costs challenging. Finally we note, we have made use of the best available data on occupations linked to wages; this necessarily excludes informal work and work in kind e.g. as a housewife or househusband.

While we have successfully attributed much of our results to node degree, we expect that our results would differ if our method were repeated in a real contact network reflecting transitivity and hierarchical organisational structure. While the role of empirical structure in network spreading is non-trivial, it is likely that the global spread would be slower^72^ than observed in our configuration model. As such we should consider the rate and depth of epidemic spread reported here to be a lower bound.

A more precise critique is that our configuration model connects edge stubs between nodes at random, once a degree has been assigned based on occupation. It might be expected that the edge structure in an empirical contact network due to organisational structure or other heterogeneities might lead to very different behaviour than those we have observed. Shuffling the edges of our contact networks i.e. an Erdos-Renyi network with equivalent average edge density, we confirm that considerable structure present in our modified configuration model is destroyed. This can be seen by comparing the distribution of various centrality measures (see SI). Likewise our benchmark empirical contact networks exhibit comparable behaviour. The finding that the function of a network is to large extent determined by the node degree sequence, and less by the precise connections is consistent with findings on the controllability of a large corpus of networks^68^.

An additional caveat relates to the inherent difficulty of assessing the full economic effect of workers being unable to work. While such epidemic containment policies are generally considered on the basis of cities or states, industries are dynamically linked through national economies and global supply chains. The full economic and social cost of epidemics and associated non-pharmaceutical interventions is so complex to model as to be out of scope of this single paper.

It is also certainly true that social networks are not static^73^. However there is no consensus on how social ties change during lockdowns and certainly not on how this is related to occupations which is the main focus of our paper. We believe that the abrupt, disruptive and heterogeneous nature of the COVID containment measures are likely to be difficult to model in a consistent way. For example, previous work has shown that the abrupt and short trauma of a natural disaster can lead to an increase in future social ties among a student population^74^. However the prolonged, and for the majority of the population of high income countries, relatively less traumatic nature of the COVID pandemic it is unclear how social networks and contact networks might each be affected. It could be argued that since many social contacts are at least partially maintained at a distance e.g. through phone calls, social media, video chat; that these contacts would not be greatly disrupted. Yet it is important to distinguish between social network ties (friendships) and contact network ties (physical interactions); our interest here is not in social networks so much as contact networks; these are not trivially related^36^ Likewise, various occupations work together in workplaces or within supply chains to produce goods at different regional, and even global, scales. Thus, the productivity of a worker is never truly fully independent of another. Practically this means that furloughing one worker could lead to another being effectively furloughed and in this case our estimate of economic cost would represent an underestimate.

Subject to the limitations noted above, our findings may have important implications for the practical implementation of epidemic control. Public health services have a limited number of tools available for epidemic containment, primarily furloughing of workers, contact tracing of identified infected individuals and pre-emptive surveillance of citizens. The precise implementations of these strategies require careful trade-offs between economic, social and privacy costs.

Our findings make the case for better data on contact patterns broken down by occupation, as well as thorough classification of occupations and workplaces in terms of their ability to incorporate mitigating strategies e.g. Personal Protective Equipment and frequent testing. However we demonstrate that a policy of furloughing workers based on total number of contacts is far more effective at minimising epidemic spread and the cost of furloughing workers as compared to other heuristics such as furloughing based upon essentialness or furloughing a proportion of the workforce at random. However we find that there are diminishing returns to increased knowledge of the contact network beyond degree. Heuristics such as centrality or control nodes are not able to significantly outperform a simple count of contact degree. This is despite a huge increase in both the amount of invasive personal data required to reconstruct a full contact network and the complexity of its collection. Practically speaking, worker degree could be straightforwardly estimated using Bluetooth proximity to other phones, or GPS co-location. Each contact need not be identified beyond a unique untraceable ID to uniquely identify repeated contacts and no further information need be shared between individual devices or with a centralised authority.

The COVID-19 epidemic has caused many profound societal changes that are unlikely to be reversed even once the disease abates due to mass vaccination. This includes scrutinisation of the nature of work in light of vast changes in demand across sectors, variable infection across occupations, the large-scale adoption of remote working and challenging deeply ingrained understandings of workplaces.

The ability to successfully automate the skills of workers, often known as skills based technological change, depends not only on overcoming the underlying physical or engineering challenges. Moreover the automating technology must be economical and must be socially and politically acceptable in order to successfully replace human labour. The changes put in motion by the COVID-19 pandemic are likely to drastically alter these considerations, in some cases leading to ‘automation forcing’^11^.

Presently ‘goods producing’ jobs that can be performed in isolation e.g. construction workers or truck drivers are considered to be at high risk of automation. In contrast ‘service producing’ jobs with a high degree of personal contact e.g. fitness instructors or nannies, are at relatively lower risk. Consequently an increase in employment share has been observed in the latter category^75^.

We have demonstrated that the nature and number of connections between workers; the structure of the contact network, has vastly more potential to effectively stop epidemic spread than a single attribute of each occupation such as wage or essentialness. Looking to the future, we might reasonably expect that research and development of automation technologies will refocus to target the skills present in occupations with a high degree of contact with others. This could in turn lead to a change in these prevailing dynamics within low wage jobs.

## Supplementary Information

Supplementary Information.
