# Supplementary material for: The network limits of infectious disease control via occupation-based targeting
Source: Sci Rep. 2021 Nov 24;11:22855. doi: 10.1038/s41598-021-02226-x (PMC8613398; doi:10.1038/s41598-021-02226-x)
Supplement: Supplementary file 1 — Supplementary Information. [file 41598_2021_2226_MOESM1_ESM.pdf]

Supplementary Information: The network limits of infectious disease control via occupation-based targeting

Demetris Avraam[1,2], Nick Obradovich[1], Niccolò Pescetelli[1], Manuel Cebrian[1,*], and Alex Rutherford[1,*]

[1]Centre for Humans and Machines, Max Planck Institute for Human Development, Berlin, Germany
[2]Population Health Sciences Institute, Newcastle University, Newcastle, UK
[*]Corresponding authors: Alex Rutherford (rutherford@mpib-berlin.mpg.de) & Manuel Cebrian (cebrian@mpib-berlin.mpg.de)

Occupational Proximity

We consider five relevant dimensions of proximity to others and infection risk, by occupation taken from the O*NET database.

1. Exposed to disease or infections[1]
2. Performing for or working directly with the public[2]
3. Communicating with persons outside of the organisation[3]
4. Deal with external customers[4]
5. Physical proximity[5]

Several of these dimensions include both a 'level' and 'importance'. Since these are in general almost collinear, we just consider only the level measure. The value of the level measures ranges from 0 to 100. Less than 2% of values were missing and were imputed with the mean value.

Given that O*NET does not provide an explicit measure of physical contacts, we strive to proxy the degree of contact with others from the measures listed above. It is expected that the metrics above are neither perfectly orthogonal nor able to completely determine the desired measure of physical contact, for example *communication with persons outside of the organisation* could be conducted via phone and not only in person. We therefore construct a composite measure of proximity from these five dimensions using a Principal Component Analysis (PCA), after subtracting the mean (the data are already standardised into a scale 0-100). This is able to explain 53% of the variance. Figure S1 shows the projection of occupations on the first and second principal components (PC).

In order to validate this approach, we compare the occupations that are assigned a high score via our method to those which have been reported to have high rates of infection[6].

Generally, comparing both barplots in Figure S2, we find that similar occupations with high proximity correlates to high death rates of related jobs during the COVID-19 epidemic, although the complexity of factors that determine real death rates is way beyond physical contacts. We note that we are comparing data from the US (O*NET) and the UK (Sky News Survey) as we are unable to find similar data for the US. However, we consider these economies to be suitably similar to offer a general comparison.

Contact Degree

We derive contact degree distributions as follows. We fit log-normal distributions to several contact degree distributions from the literature[8–13]. For each fit, we get the mean and the variance and calculate the Coefficient of Variation (CV) which is given as below for a log-normal distribution X:

$$E(X) = \exp(\mu + \sigma^2/2),$$

$$var(X) = (\exp(\theta\sigma^2) - 1) \times \exp(2\mu + \sigma^2\theta),$$

$$CV(X) = \sqrt{var(X)}/E(X) = \sqrt{(\exp(\theta\sigma^2) - 1)}$$

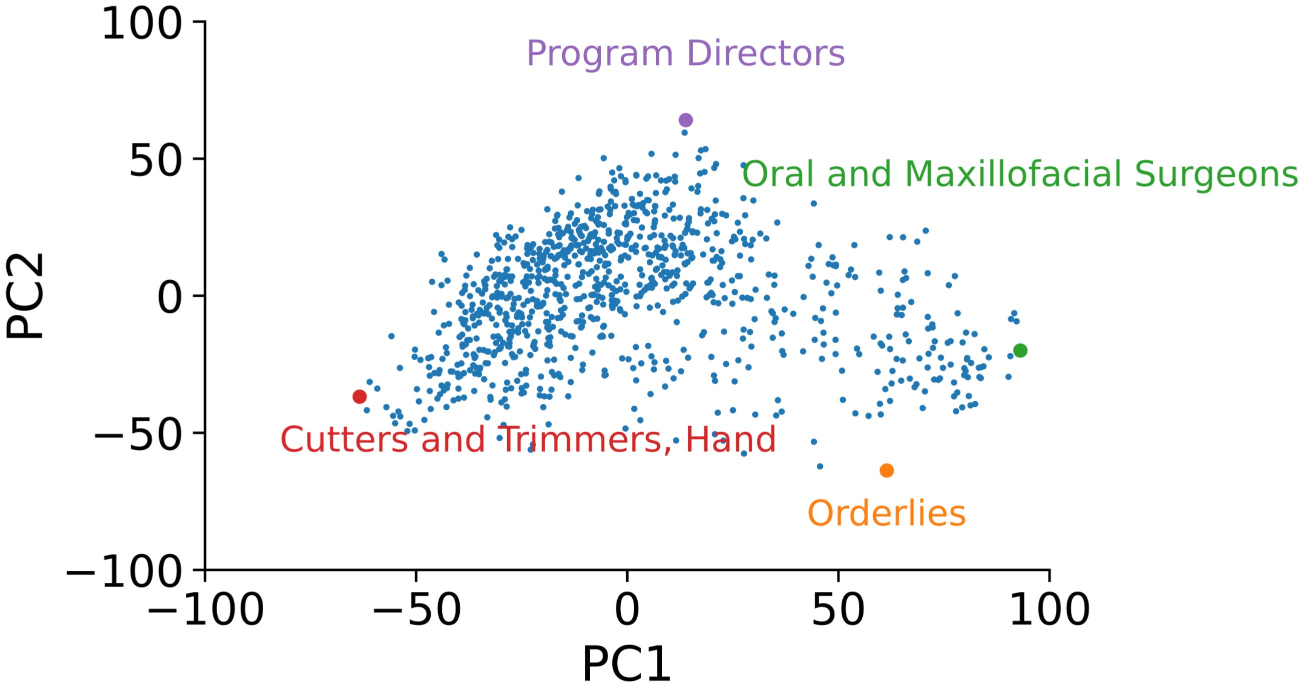

Figure S1. Projection of selected jobs on the first and second principal components (PC).

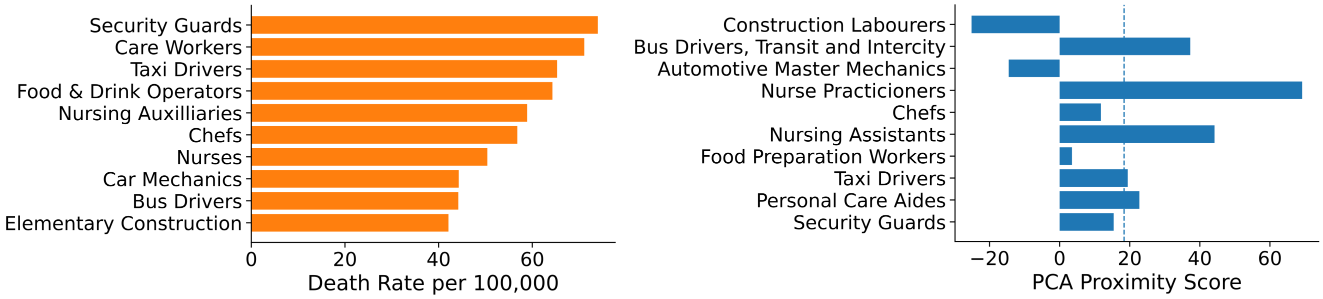

Figure S2. The COVID-19 death rate in top ten jobs as reported in[7] (left) and the composite proximity score for the closest matching occupations in the O*NET data.

where μ and σ^2 are the mean and the variance of the normal distribution $Y = log(X)$.

The parameters of each fit are listed in Table S1. We wish to recover the mean number of contacts of 75 as observed in[14] and to split these total contacts into work, home and transport (more accurately representing all other urban interactions: transport but also shops, cinemas, restaurants etc) while imposing a skew that is consistent with the Coefficient of Variation observed empirically. We choose a CV value of 0.7 representative of the mean and the middle of this range of CV values.

Home: the mean home degree is derived from census information[15] which gives a mean of 2. The standard deviation (SD) is derived from the CV=0.7. We sample from this distribution independently of occupation for each worker.

Work: the work degree is derived by matching the percentiles of (i) the PCA score described above and (ii) a log-normal distribution with mean 36.5[14]. The SD is derived from the CV=0.7. The work degree is fixed between workers with the same occupation.

Transport: the mean transport degree of 36.5, is given by the remaining contact degree after accounting for home and work. Once again the SD is derived from the CV. Transport degree is sampled from this distribution for each work independent of occupation.

	$\mu = E(Y)$	$\sigma = \sqrt{var(Y)}$	$E(X)$	$\sqrt{var(X)}$	$CV(X)$
Workplace	3.55	0.61	42.05	28.50	0.68
Conference	3.62	0.77	50.37	45.39	0.90
Art exhibition	2.36	0.78	14.34	13.13	0.92
Hospital	3.27	0.57	30.91	19.20	0.62
Primary school	4.15	0.43	69.21	30.82	0.45
High school	3.07	0.58	25.51	15.98	0.63

Table S1. Parameters of log-normal distribution fitted to the degree distribution of six empirical networks.

Occupational Proximity to Contact Degree

As described in the main paper, we have a multidimensional score of proximity in the range [0-100] for each occupation. We seek to translate that score into a work contact.

These distributions are fitted to data found in[16]. The values are then rescaled to make a mean total of 75, matching empirical data[14].

The work degree of the node depends solely on her occupation, which is proportional to the rescaled value of its projection on the first PC of the job proximity data with the mean work degree equal to 36.5.

We also calculate the correlation between the first PC with the essential score data found in[17,18] (see Figure S3).

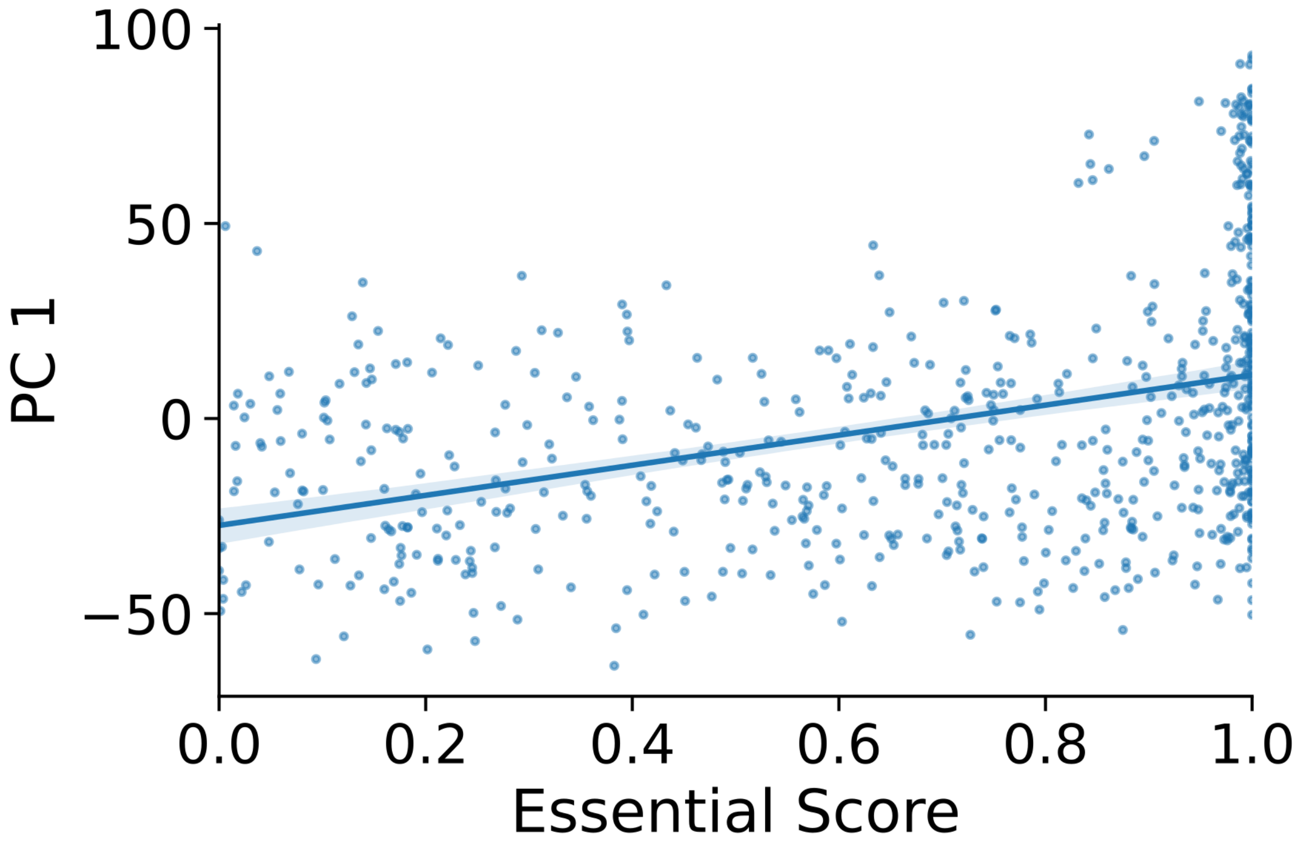

Figure S3. Essential score from Rio-Chanona et al vs first Principal Component of proximity data. Pearson correlation is 0.375.

Workforce Data

To generate the occupational contact network through the configuration model, we use data from the NY Workforce dataset for 2019[19] and the data for the number of contacts per occupation that we derive through the PCA. The NY workforce dataset includes information about the 750 distinct occupations that exist in the NY, such as the number of employees per occupation (out of a total labour size of around 9.5M workers), their percentage in the NY workforce, and the hourly and annual average wages per worker per occupation. Each occupation in the dataset is represented by a six-digit code based on the 2010 Standard Occupational Classification (SOC) System[20]. The first two digits represent the major group, the third digit represents the minor group, the fourth and fifth digits represent the broad occupation and the sixth digit represents the broader occupation. The top three occupations by number of employees in the NY workforce, are the Retail Salespersons (SOC:41-2031), the Home Health Aides (SOC:31-1011) and the Cashiers (SOC:41-2011) with 302430, 202660 and 198980 employees respectively (that is around 3.2%, 2.1% and 2.1% of the NY workforce). The bottom three occupations in the dataset are the Fallers (SOC:45-4021), Radio Operators (SOC:27-4013) and Hoist and Winch Operators (SOC:53-7041) with just 30, 40 and 40 workers respectively.

Merging O*NET and Workforce Data

The data for the number of contacts per occupation derived by the PCA include information for 967 occupations. The occupations in this dataset are represented by an O*NET-SOC eight-digit code. The first six digits match the SOC coding scheme while the additional two digits indicate whether the O*NET-SOC occupation is a one-to-one match or is a detailed O*NET breakout of the SOC occupation[21]. To merge the two datasets by occupation code, we first group the occupations included in the PCA outcomes by their six first SOC digits. In that case, the number of contacts per six-digit SOC occupation is the mean of the number of contacts between the eight-digit SOC occupations that share the first six digits. After grouping the occupations (i.e. transition from the eight-digit coding system to the six-digit classification), we end up with 773 distinct occupations. 680 out of 750 SOC codes from the NY workforce dataset were matched one-to-one with SOC codes from the contacts per occupation data. We then match 18 more occupations presented in Table S2 manually. We therefore create a dataset of 678 distinct occupations having their number of employees in the NY workforce, their average wages and the number of contacts per occupation.

Modified Configuration Model

We generate our contact network between workers using a modified configuration model as follows. We first represent the assigned numbers of home, work and transportation contacts of each worker as half-links of the nodes in the network (see Figure 2 in the main paper). We then randomly select a pair of half links for each category of contacts and connect them. The random selection is achieved by sampling nodes without replacement, in order to avoid duplications of contacts and self-loops. We repeat the process until all half-links are paired up. This technique produces a network that is not fully random as nodes are assigned a degree according to their occupation which is in turn taken from an empirical distribution of workers over occupations.

Calculation of Transmission Rate from Reproductive Number

Let us consider how we can relate R_0, the mean number of new infections per infection, to p_{inf} and $\langle d \rangle$; the probability of infection between two nodes in a single encounter and the mean network degree. Since $R_0 \approx 2.5$ according to various studies, we would like to fix p_{inf} appropriately in our simulations to be roughly consistent with this measured R_0.

Let us consider a 'point infection' in a single time-step (in our simulations this is a single day) in which node i interacts with its four neighbours (see Figure S4). With probability p_{inf} node j is infected by node i and transitions from susceptible to infected.

In a single encounter the probability of j becoming infected is $p_j(S \to I) = p_{inf}$. Under n encounters, the probability of j becoming infected in at least one of the n encounters is the complement of the probability that j is not infected in any of the n encounters. The probability of not becoming infected in a single encounter is $(1 - p_{inf})$.

$$p_j^{n,p_{inf}}(S \to I) = (1 - (1 - p_{inf})^n)$$

This can be expanded to an arbitrary number of neighbours (see Figure S5). Assuming that there are no spillovers e.g. there is no possibility for i to infect k and then k to subsequently infect j (with i and j connected), we can calculate the expected number of new infections as the product of this probability of infection after n days and the mean degree. This is our implied R_0. This assumption of no second order effects is somewhat unrealistic and becomes more unrealistic as the incubation period increases.

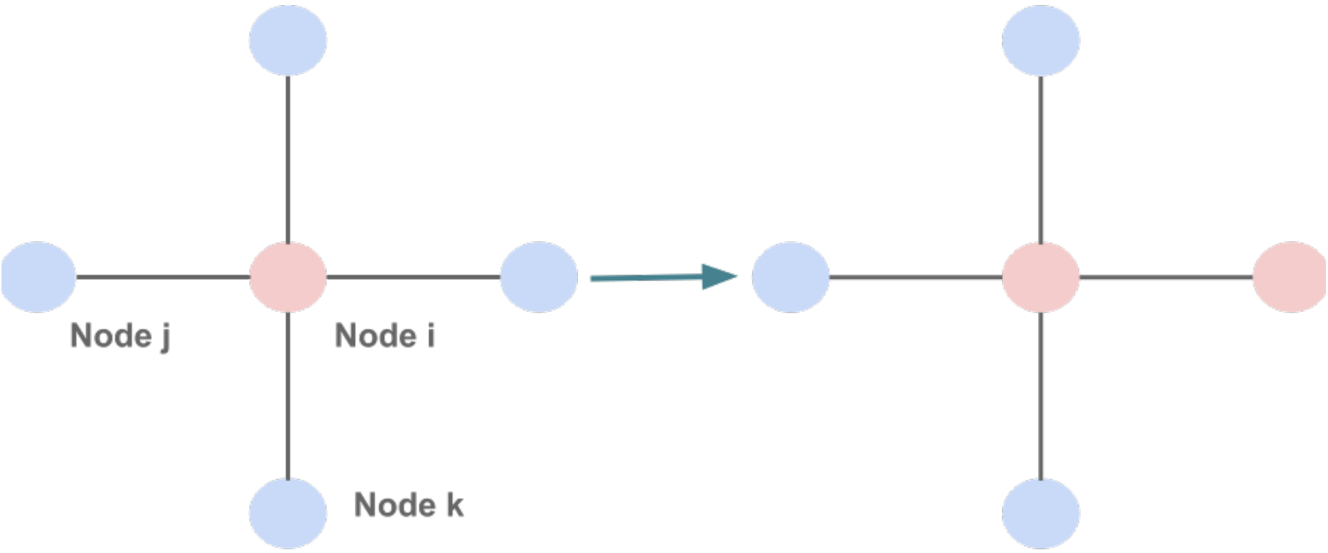

Figure S4. A schematic illustration of a contact infection transmission.

$$R_0^{implied} = \langle d \rangle \times p_j^{n,p_{inf}}(S \rightarrow I)$$

$$R_0^{implied} = \langle d \rangle \times (1 - (1 - p_{inf})^n)$$

Assuming that R_0 is defined over the incubation period i.e. it is the mean number of new infections caused by an infected person before they are symptomatic, then we set n = 14. $\langle d \rangle$ is defined by our contact data and so p_{inf} is set by our empirical R_0.

In fact we can calculate p_{inf} directly, given our assumptions. Given our assumption of no higher order effects (i infects k who infects j) we would actually need a smaller p_{inf} to recover R_0. So our estimated value of p_{inf} is an upper bound.

$$p_{inf} = 1 - \sqrt[n]{1 - R_0/\langle d \rangle} \sim 0.0024$$

Industry Based Worker Productivity

To examine the productivity of workers based upon the industry of their occupation, we employ data from the Bureau of Economic Analysis (BEA) on value added by industry in producers' prices[22]. These data are calculated at the detailed industry level and exist for the years 2007 and 2012. We employ the most recent set of these data – from 2012. We note that while industry value added has surely changed since these data were compiled, the detail industry values in BEA's estimates likely correlate decently well with actual present day value added and provide to our knowledge the highest resolution estimate of detailed industry value added in the U.S.

The BEA industry codes (NAICS) do not perfectly match the industry codes contained within the O*NET data, and so we supplement the NAICS crosswalk provided by the U.S. Census[23] with a procedure that sequentially matches industries to occupations based upon increasing levels of industry aggregation. We first match industry value added data on the full six digit NAICS codes of occupations, then match any remaining occupations and industries at the five-digit level, and so on up to the two-digit NAICS code level. Approximately 27% of occupations have a full match, 0.3% have a five digit match, 15% have a four digit match, 28% have a three digit match, and 29% have a two digit match.

Evaluation of Strategies

In order to evaluate and compare strategies, we consider (i) the economic loss due to furloughing of workers (ii) the economic loss due to infected workers and (iii) the size of the peak of the number of infected individuals (as a proxy for the degree of strain on health services). We wish to evaluate each strategy independently from a specific choice about the severity of the

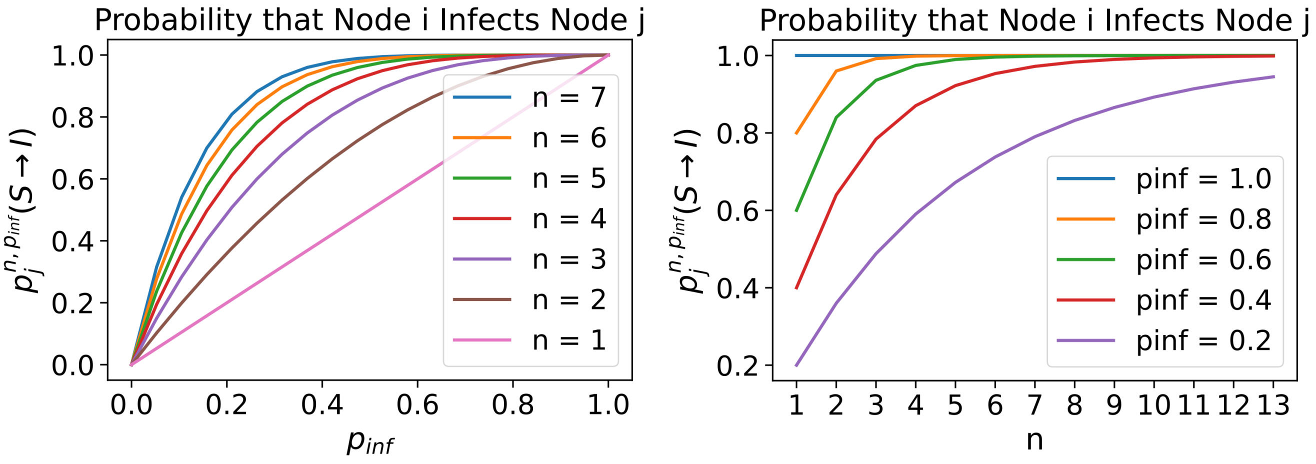

Figure S5. The probability of a node to become infected based on the number of its infected neighbours (left) and the probability of infection in a single encounter (right).

intervention i.e. the proportion of workers to furlough. We therefore calculate the areas under the curves plotted in Figure 4 of the main paper; higher values are worse for all metrics. This is a simple sum given as below for strategy s for the furlough loss and analogously for loss due to infection and epidemic peak.

$$AUC^{s}_{furlough} = \sum_{n_{percent}} furloughloss^{s}(n_{percent})$$

In Figure S6, we present the interaction term between the cost due to furloughing and the cost of infection that complements Figure 5 in the main paper.

Centrality and Shuffled Centrality

Assigning a score to each node in a network according to its centrality is a mature area of study. We therefore compare various centrality measures (see Table S3 and Figure S7), we conclude that High Degree Adaptive (HDA) is the best performing and it is this metric we present in the main paper.

Decomposition of the network in subcomponents

Our initial network is composed of a single component with 200,003 nodes. When removing the work and transportation links of nodes that can work from home the network decomposes into 3,846 components where from those, one is the giant component with 194,276 nodes and most of the other components are isolated nodes reflecting the workers that can work from home and do not have any home links. Furloughing n% nodes additional to those that can work from home, the network further decomposes in more components and the size of the giant component reduces as shown in Figures S8 and S9 for the different furloughing strategies.

Proximity and Other Worker Measures

Figure S10 shows the weak correlation between occupational proximity and essentialness score (left) and between occupational proximity and wage (right).

Comparison of Synthetic Contact Network with Empirical Contact Networks

We address the concern that our results are an artefact of our contact network generation model; the modified configuration model. Therefore we consider several empirical contact networks from various scenarios and compare the descriptive statistics of each with our synthetic network (table S4 and Figure S11). The “workplace” is the contact network between 217 individuals measured in an office building in France in 2015. The “conference” network represents the face-to-face interactions of 403 participants to the 2009 SFHH conference in Nice, France. The “art exhibition” is the network of contacts collected during an

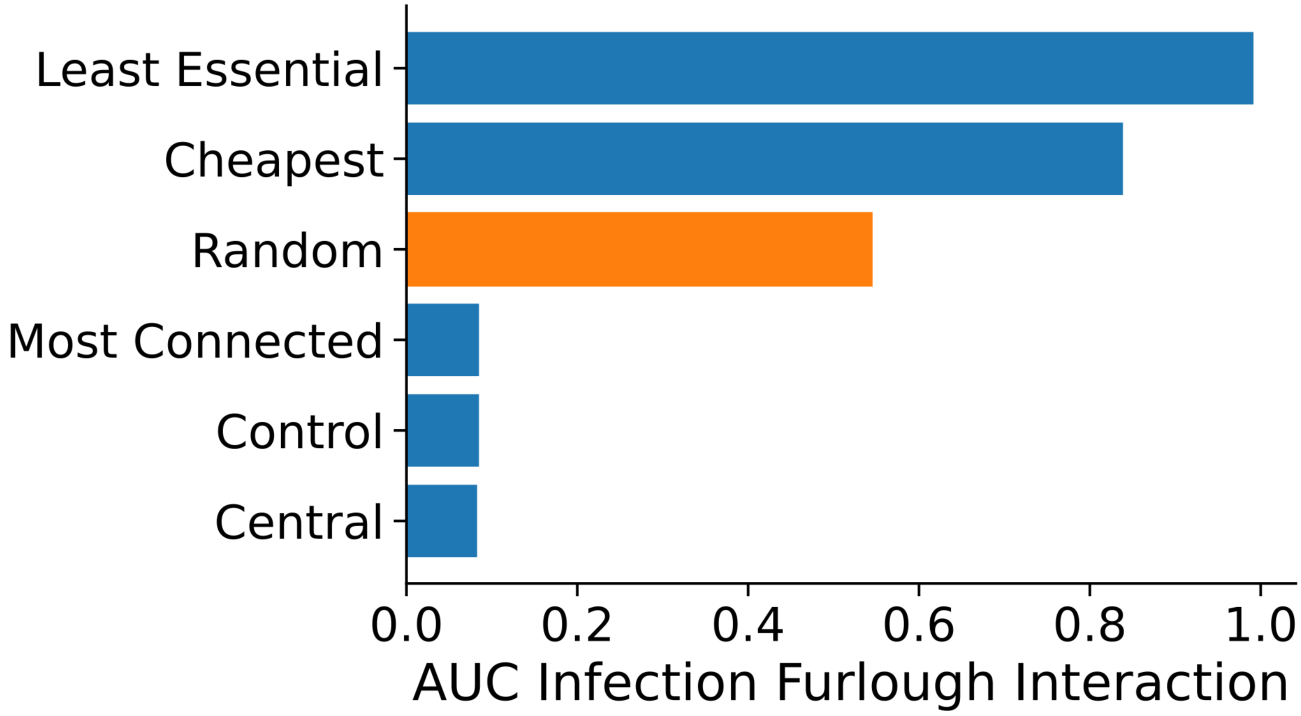

Figure S6. The aggregated performance of each strategy presented as the interaction term between the cost due to furloughing and the cost of infection that complements Figure 5 in the main paper.

art science exhibition at the Science Gallery in Dublin, Ireland in 2009. The "hospital" is the contact network between patients, between health-care workers and patients and among health-care workers during four days in a hospital ward in Lyon, France. The "primary school" network includes the face-to-face interactions between 242 students and teachers in a primary school, and the "high school" is the network of contacts between students in a high school in Marseilles, France. The data for those networks were active contacts as collected during 20-second intervals generating dynamic (temporal) networks in the different locations. Here we consider their static counterparts by simplifying the networks with neglection of multiple edges or by using cumulated contacts (e.g. within a day).

Simulations with random rewiring

We randomly delete 1% of home, transportation and work edges respectively and then rewire random nodes with the same proportion of links. We then repeat the simulations with the furloughing strategies on that network, and compare the outcomes (Figure S12) with the main findings shown in Figure 4 of the main paper. Each spreading scenario shown in Figure S12, is evaluated over 1000 epidemic realisations.

Simulations with additional removal of home links of the furloughed workers

We repeat the simulations with the different furloughing strategies and we add the hypothesis of home quarantine (Figures S13 and S14), which means that for the furlough nodes we cut all of their links (work, transportation and home).

Comparison with Alternate Configuration Model Instantiation

In order to ensure that our results are not an artifact of our particular random configuration of the network, we repeat our analysis using a different random seed. The results of the analysis with the different network configuration are shown in Figure S15.

References

1. Work context: Exposed to disease or infections. https://www.onetonline.org/find/descriptor/result/4.C.2.c.1.b?a=1. Accessed: 2020-10-17.

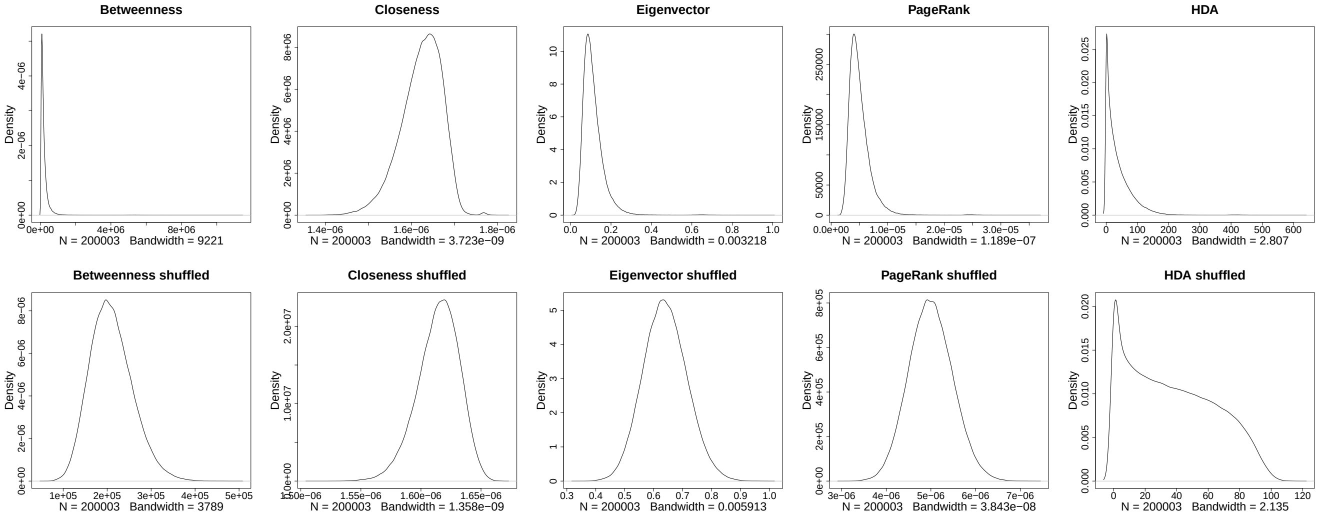

Figure S7. Distribution (kernel density) of centrality values for our contact network constructed using the modified configuration model (top row) and under edge shuffling (i.e. an Erdos Renyi network with equivalent size and edge density).

2. Work activities: Performing for or working directly with the public. https://www.onetonline.org/find/descriptor/result/4.A.4.a.8. Accessed: 2020-10-17.
3. Work activities: Communicating with persons outside organization. https://www.onetonline.org/find/descriptor/result/4.A.4.a.3. Accessed: 2020-10-17.
4. Work context: Deal with external customers. https://www.onetonline.org/find/descriptor/result/4.C.1.b.1.f. Accessed: 2020-10-17.
5. Work context: Physical proximity. https://www.onetonline.org/find/descriptor/result/4.C.2.a.3. Accessed: 2020-10-17.
6. Coronavirus: Male security guards, chefs and taxi drivers among those most likely to die with COVID-19, says ONS. https://news.sky.com/story/coronavirus-male-security-guards-chefs-and-taxi-drivers-among-those-most-likely-to-die-with-covid-19-says-ons-11986382 (2020). Sky News, Accessed: 2020-10-17.
7. Coronavirus: Security guards are most at risk of dying with COVID-19, figures show. https://news.sky.com/story/coronavirus-security-guards-are-most-at-risk-of-dying-with-covid-19-figures-show-12015241 (2020). Sky News, Accessed: 2020-10-17.
8. Stehlé, J. *et al.* High-Resolution Measurements of Face-to-Face Contact Patterns in a Primary School. *PLoS ONE* **6**, e23176, DOI: https://doi.org/10.1371/journal.pone.0023176 (2011).
9. Cattuto, C. *et al.* Dynamics of Person-to-Person Interactions from Distributed RFID Sensor Networks. *PLoS ONE* **5**, e11596, DOI: https://doi.org/10.1371/journal.pone.0011596 (2010).
10. Génois, M. & Barrat, A. Can co-location be used as a proxy for face-to-face contacts? *EPJ Data Sci.* **7**, DOI: https://doi.org/10.1140/epjds/s13688-018-0140-1 (2018).
11. Génois, M. *et al.* Data on face-to-face contacts in an office building suggest a low-cost vaccination strategy based on community linkers. *Netw. Sci.* **3**, 326–347, DOI: http://doi.org/10.1017/nws.2015.10 (2015).
12. Stehlé, J. *et al.* Simulation of an SEIR infectious disease model on the dynamic contact network of conference attendees. *BMC Med.* **9**, 87, DOI: https://doi.org/10.1186/1741-7015-9-87 (2011).
13. Isella, L. *et al.* What's in a crowd? Analysis of face-to-face behavioral networks. *J. Theor. Biol.* **271**, 166–180, DOI: https://doi.org/10.1016/j.jtbi.2010.11.033 (2011).
14. Bakker, M., Berke, A., Groh, M., Pentland, A. & Moro, E. Effect of social distancing measures in the New York City metropolitan area. https://connection.mit.edu/sites/default/files/publication-pdfs/Effect%20of%20social%20distance%20measures%20in%20social%20distancing%20in%20the%20NY%20area.pdf (2020). Boston: Massachusetts Institute of Technology, Accessed: 2021-05-10.
15. Ellen, I. G. & O'Flaherty, B. Social programs and household size: evidence from New York city. *Popul. Res. Policy Rev.* **26**, 387–409, DOI: https://doi.org/10.1007/s11113-007-9036-7 (2007).

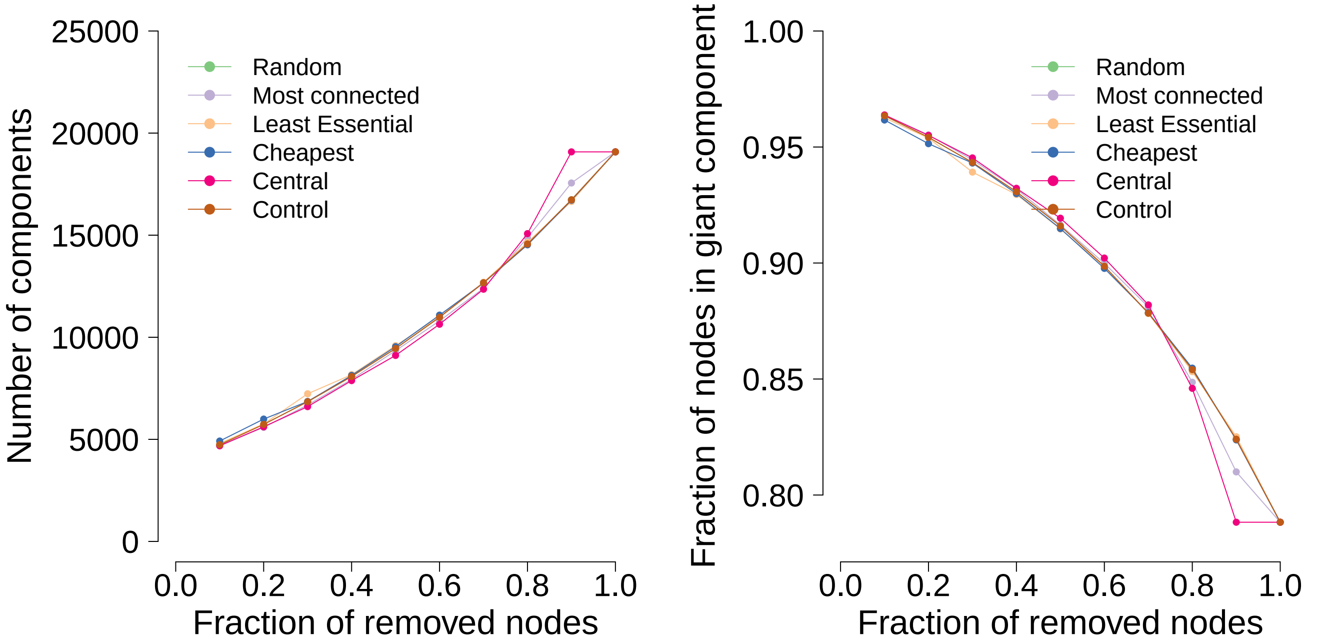

Figure S8. Left: number of clusters in the contact network as we remove the work and transportation links of a fraction of nodes from the network. Right: the fraction of nodes in the giant component as we remove the work and transportation links of a fraction of nodes from the network.

16. SocioPatterns.org. http://www.sociopatterns.org/. Accessed: 2021-03-19.

17. del Rio-Chanona, R. M., Mealy, P., Pichler, A., Lafond, F. & Farmer, J. D. Supply and demand shocks in the COVID-19 pandemic: An industry and occupation perspective. *Oxf. Rev. Econ. Policy* **36**, S94–S137, DOI: https://doi.org/10.1093/oxrep/graa033 (2020).

18. del Rio Chanona, R. M., Mealy, P., Pichler, A., Lafond, F. & Farmer, J. D. Data accompany the paper "Supply and demand shocks in the COVID-19 pandemic: An industry and occupation perspective". https://zenodo.org/record/3751068#.YThykC0RrfY (2020). Accessed: 2020-10-16.

19. U.S. Bureau of Labor Statistics. Occupational employment statistics: May 2019 metropolitan and nonmetropolitan area occupational employment and wage estimates. https://www.bls.gov/oes/current/oes_35620.htm (2019). Accessed: 2020-10-16.

20. U.S. Bureau of Labor Statistics. 2010 soc user guide: Standard occupational classification and coding structure. https://www.bls.gov/soc/soc_2010_class_and_coding_structure.pdf. Accessed: 2020-10-16.

21. Farr, J. L. & Tippins, N. T. *Handbook of Employee Selection* (Taylor & Francis, 2017).

22. U.S. Bureau of Economic Analysis. Industry Economic Accounts. https://apps.bea.gov/industry/xls/io-annual/Use_SUT_Framework_2007_2012_DET.xlsx (2018). Accessed: 2021-01-18.

23. Census, U. S. 2017 industry code list with crosswalk. https://www2.census.gov/programs-surveys/demo/guidance/industry-occupation/2017-industry-code-list-with-crosswalk.xlsx. Accessed: 2021-01-18.

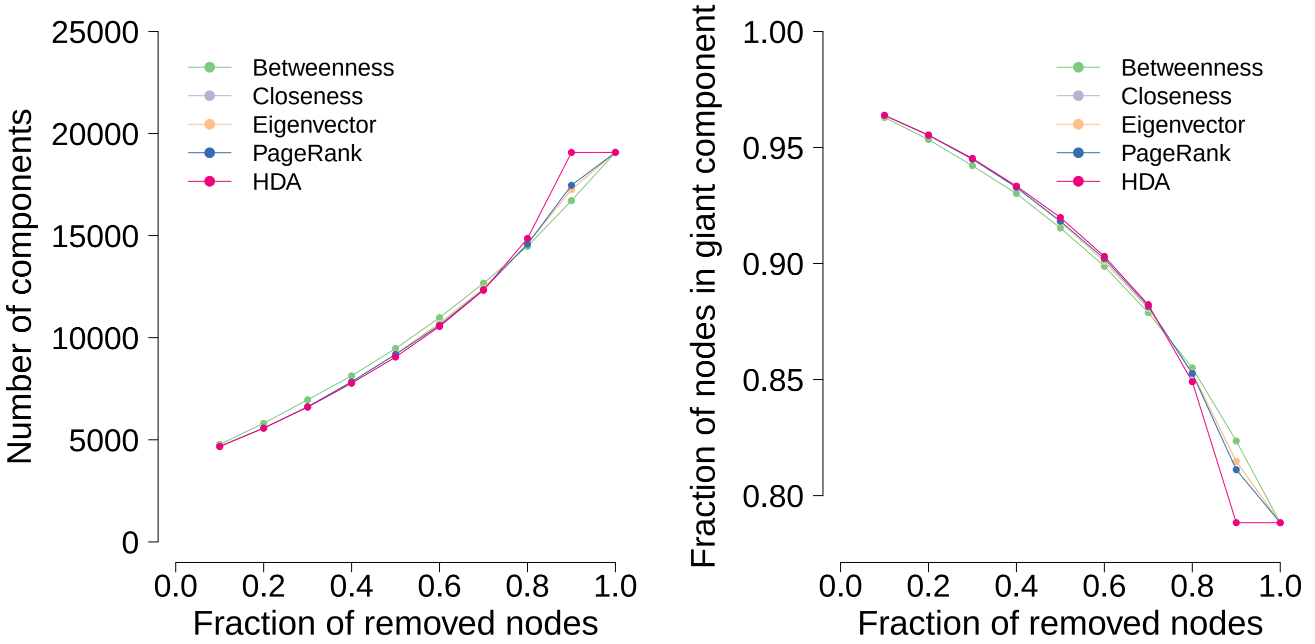

Figure S9. Comparing various centrality measures as metrics for worker removal. Left: number of clusters in the contact network as we remove the work and transportation links of a fraction of nodes from the network. Right: the fraction of nodes in the giant component as we remove the work and transportation links of a fraction of nodes from the network.

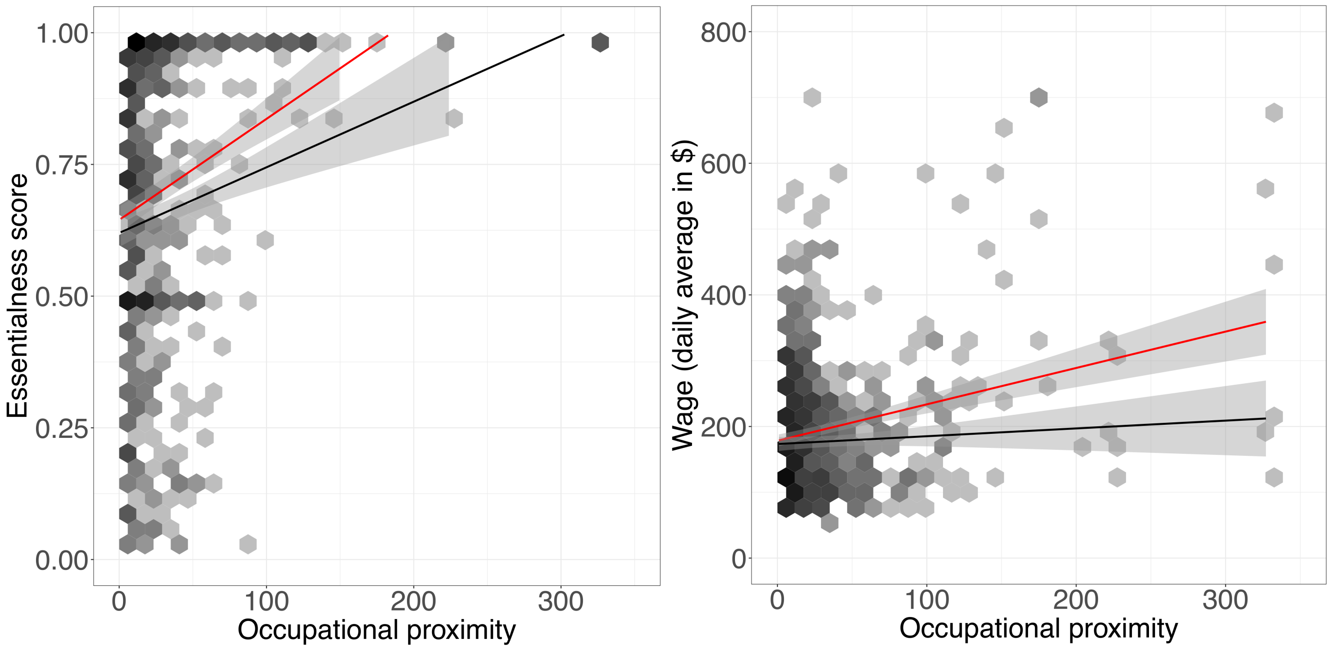

Figure S10. Hexbin plots of proximity against essentialness score (left) and wage (right) per occupation. Red line shows unweighted fit and black line shows fit weighted by the number of workers in each occupation. The essentialness score has Pearson correlation 0.29 and 0.19 when unweighted and weighted respectively, while the wage has 0.24 and 0.05 respectively.

Occ Code in NY workforce data	Occ Title in NY workforce data	Aggregated Occ Code in contacts data	Occ Title in contacts data
13-1020	Buyers and Purchasing Agents	13-1021	Buyers and Purchasing Agents, Farm Products
19-1099	Life Scientists, All Other	19-1011	Animal Scientists
21-1018	Substance Abuse, Behavioral Disorder, and Mental Health Counselors	21-1011	Substance Abuse and Behavioral Disorder Counselors
21-1019	Counselors, All Other	21-1014	Mental Health Counselors
29-2010	Clinical Laboratory Technologists and Technicians	29-2011	Medical and Clinical Laboratory Technologists; Cytotechnologists; Histotechnologists and Histologic Technicians; Cytogenetic Technologists
35-2019	Cooks, All Other	35-2013	Cooks, Private Household
39-1010	First-Line Supervisors of Gaming Workers	11-9071	Gaming Managers
39-7010	Tour and Travel Guides	39-7011	Tour Guides and Escorts
47-3019	Helpers, Construction Trades, All Other	47-3016	Helpers–Roofers
49-9069	Precision Instrument and Equipment Repairers, All Other	49-9045	Refractory Materials Repairers, Except Brickmasons
51-2028	Electrical, Electronic, and Electromechanical Assemblers, Except Coil Winders, Tapers, and Finishers	51-2022	Electrical and Electronic Equipment Assemblers
51-2098	Assemblers and Fabricators, All Other, Including Team Assemblers	51-2093	Timing Device Assemblers and Adjusters
51-4199	Metal Workers and Plastic Workers, All Other	51-4192	Layout Workers, Metal and Plastic
51-7099	Woodworkers, All Other	51-7031	Model Makers, Wood
53-1048	First-Line Supervisors of Transportation and Material Moving Workers, Except Aircraft Cargo Handling Supervisors	53-1031	First-Line Supervisors of Transportation and Material-Moving Machine and Vehicle Operators
53-6099	Transportation Workers, All Other	53-4013	Rail Yard Engineers, Dinkey Operators, and Hostlers
29-1029	Dentists, All Other Specialists	29-1024	Prosthodontists
41-9099	Sales and Related Workers, All Other	41-9091	Door-To-Door Sales Workers, News and Street Vendors, and Related Workers

Table S2. Matching unmatched SOC codes between O*NET and contacts per occupation data.

Centrality Metric	AUC Econ Loss ($B)	AUC Infected Loss ($B)
Betweenness	16.68 (± 3.15)	0.255 (± 0.006)
Closeness	11.18 (± 2.34)	0.080 (± 0.004)
Eigenvector	11.15 (± 2.36)	0.079 (± 0.004)
Page Rank	10.91 (± 2.27)	0.077 (± 0.004)
HDA	10.79 (± 2.24)	0.077 (± 0.004)

Table S3. Area under curve (AUC) of cost due to furloughing and cost due to infection under strategies based on different network centrality measures. Values indicate mean (± SD) of the AUC where each point in the curve is the average of 1000 random epidemic simulations.

Network	Nodes	Edges	Density	Min Degree	Max Degree	Average Degree	Assortativity	Transitivity	Driver nodes Switchboard (density)
Synthetic NY workforce	200003	7491270	0.0004	13	633	74.91	0.003	0.0005	95,857 (0.48)
Workplace	217	4274	0.182	1	84	39.39	0.044	0.356	99 (0.46)
Conference	403	9564	0.118	1	169	47.46	-0.081	0.236	151 (0.37)
Art exhibition	410	2765	0.033	1	50	13.49	0.226	0.436	200 (0.49)
Hospital	75	1138	0.41	6	61	30.35	-0.18	0.587	30 (0.40)
Primary school	242	8317	0.285	20	134	68.74	0.118	0.48	120 (0.50)
High school	180	2220	0.138	2	56	24.67	0.046	0.434	81 (0.45)

Table S4. Network characteristics for our occupational contact network and six empirical contact networks.

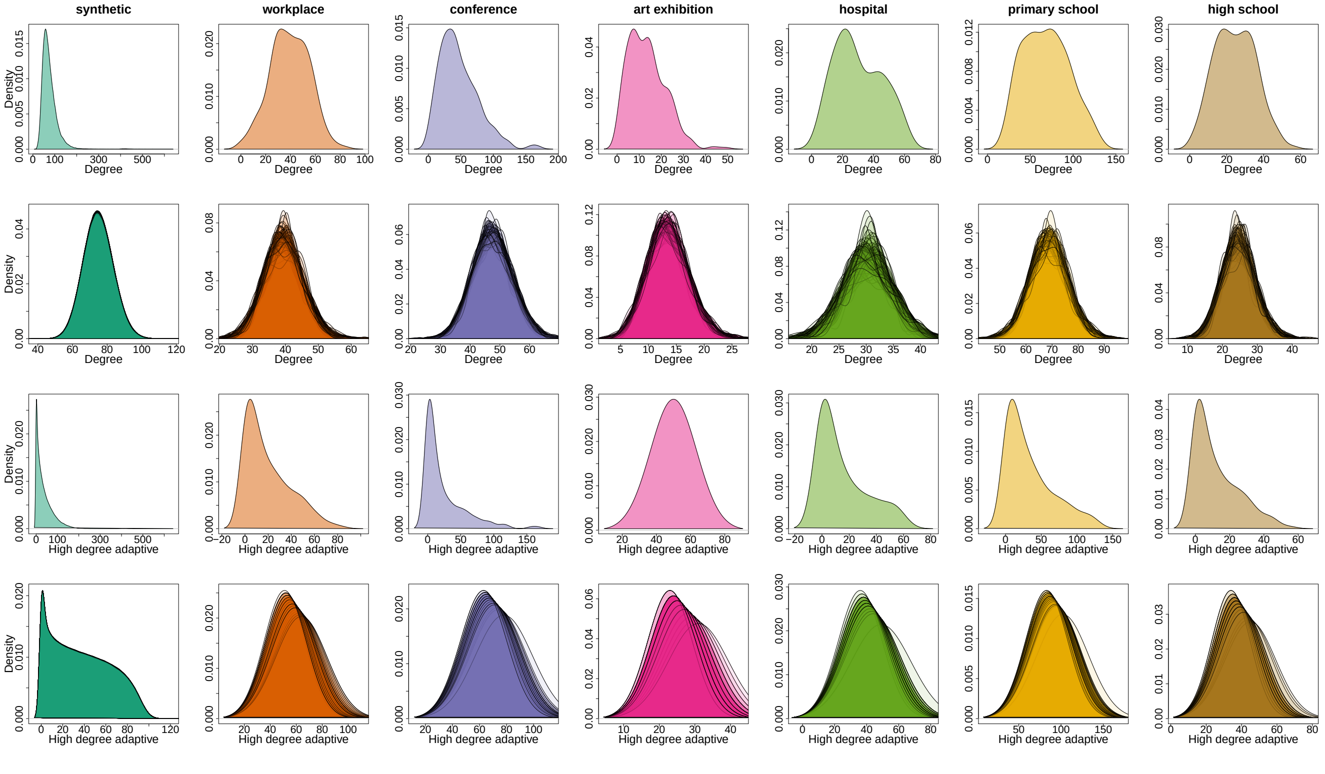

Figure S11. Comparison of distributions (kernel densities) of observed and shuffled node degrees (first and second row respectively) and distributions of observed and shuffled high degree adaptive centrality values (third and fourth rows respectively). Left hand column is our occupational network, and the remaining columns are six empirical contact networks.

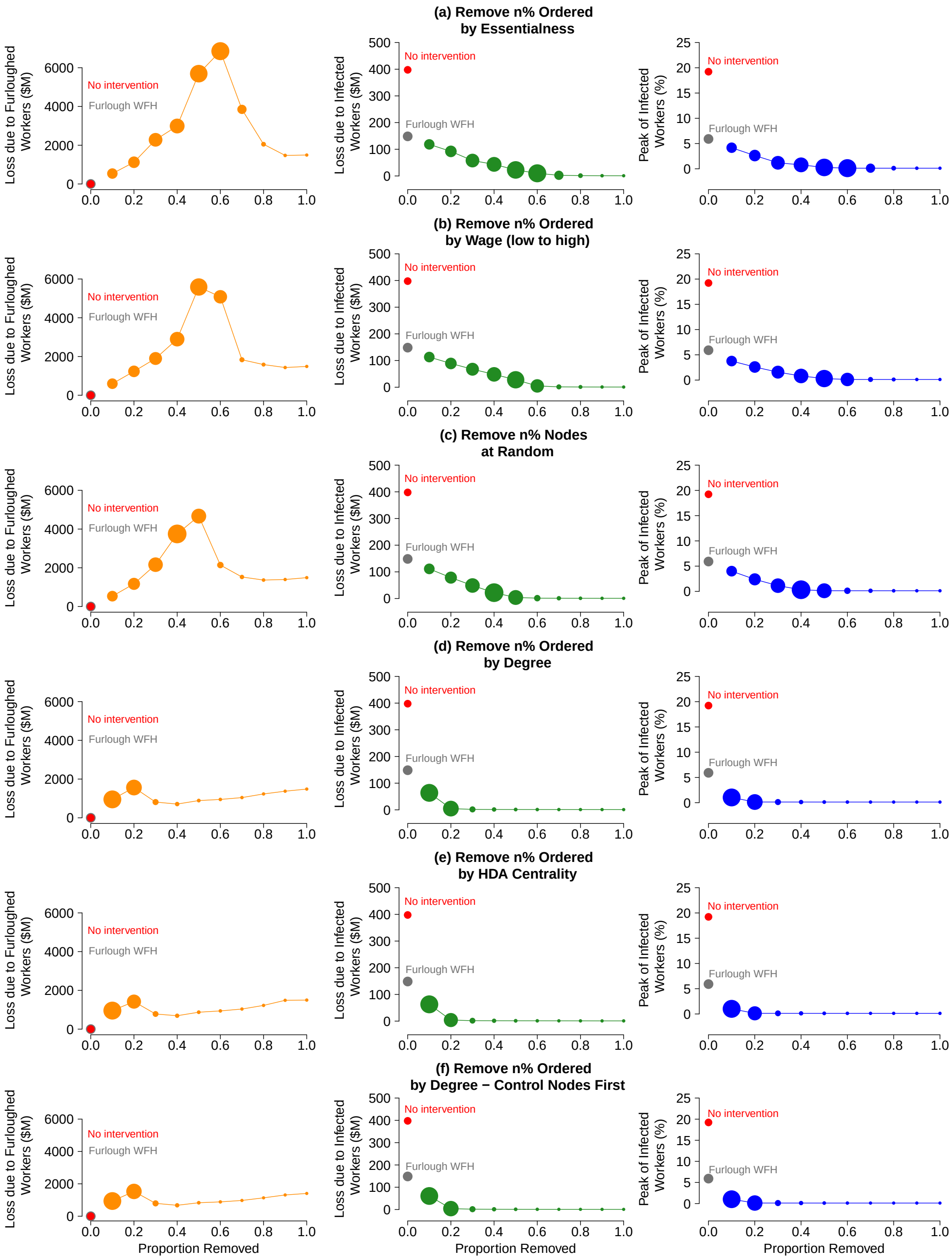

Figure S12. This figure is similar to Figure 4 in the main paper comparing the different strategies across the full range of severities according to cost of furlough (left), cost of infection (middle) and peak of infection (right). In this case the network has 1% of links randomly rewired.

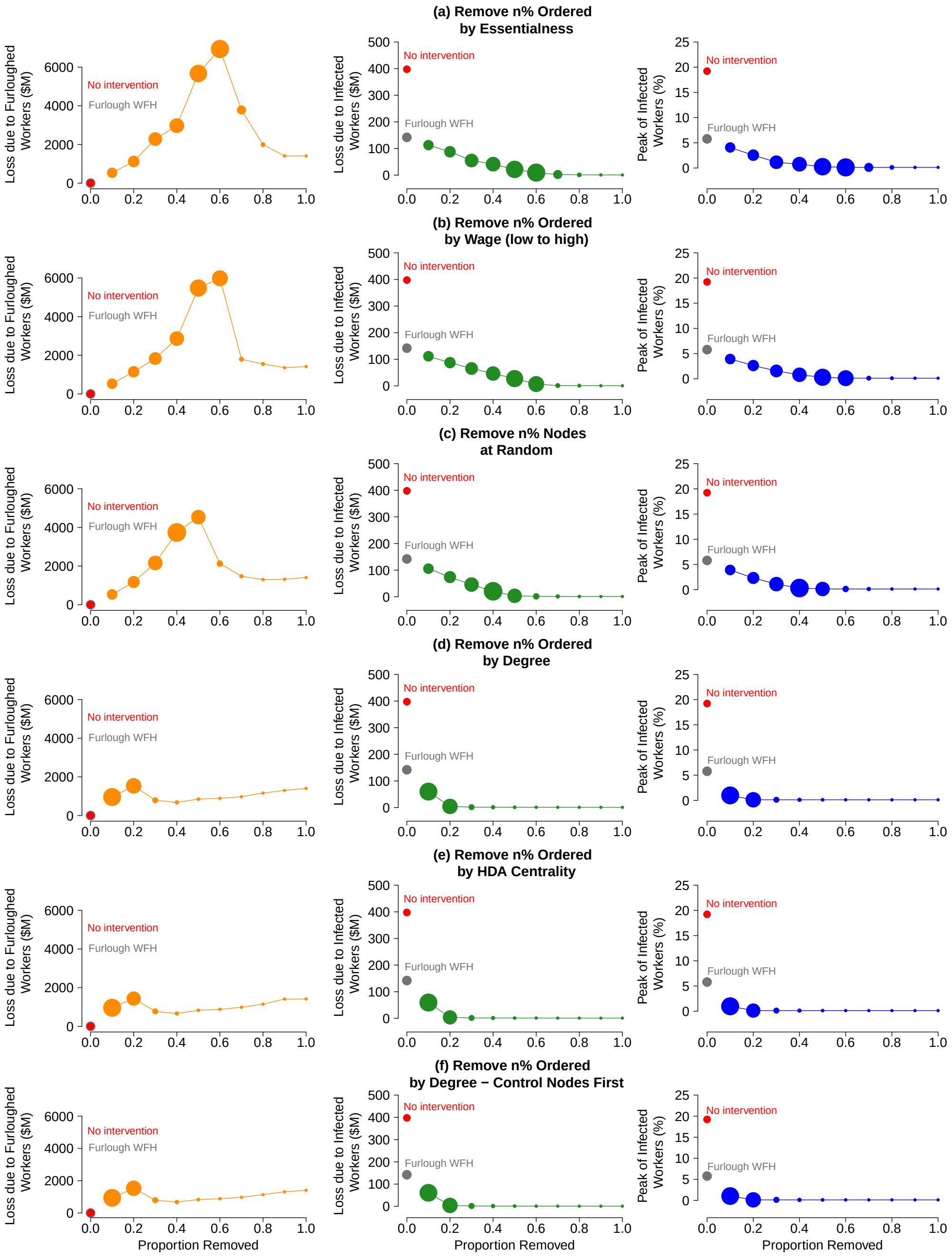

Figure S13. This figure is similar to Figure 4 in the main paper comparing the different strategies across the full range of severities according to cost of furlough (left), cost of infection (middle) and peak of infection (right). Here, for each strategy we remove the home links of furloughing workers in addition to the removal of their work and transportation links (i.e. a "home quarantine" scenario).

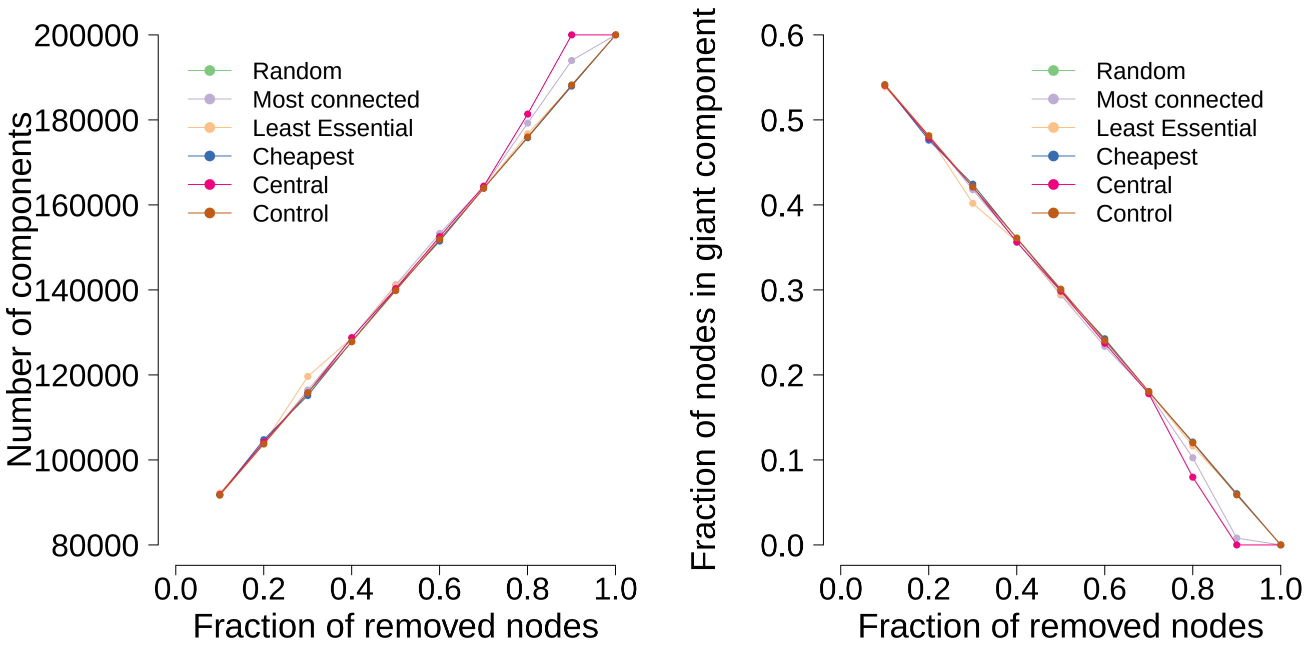

Figure S14. Left: number of clusters in the contact network as we remove a fraction of nodes from the network under the home quarantine scenarios. Right: the fraction of nodes in the giant component as we remove a fraction of nodes from the network under the home quarantine scenarios.

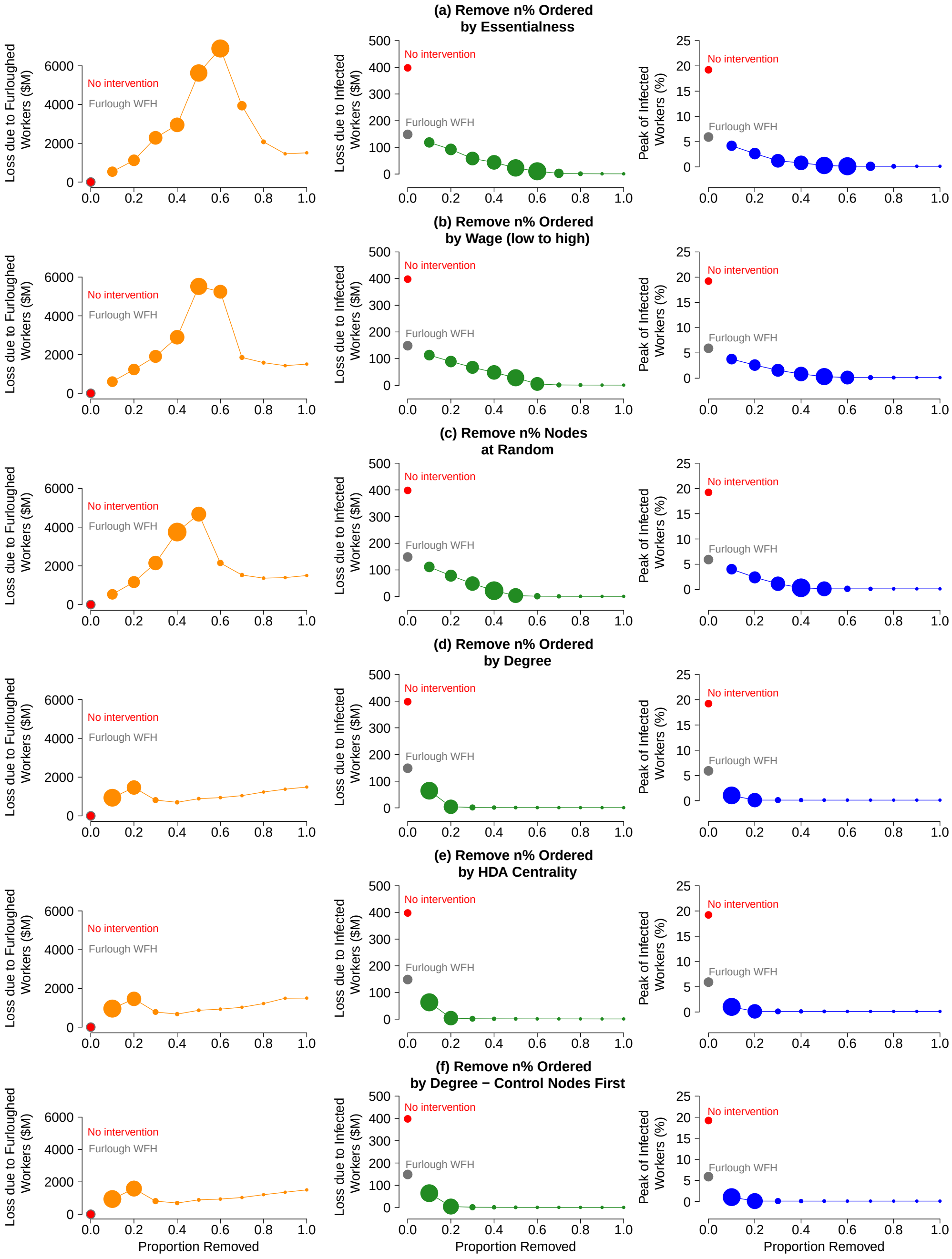

Figure S15. This figure is similar to Figure 4 in the main paper comparing the different strategies across the full range of severities according to cost of furlough (left), cost of infection (middle) and peak of infection (right). Here, we consider a different network configuration drawn from the same modified configuration model.
